# The Transcriptional Response to Lung-Targeting Lipid Nanoparticles *in Vivo*

**DOI:** 10.1021/acs.nanolett.2c04479

**Published:** 2023-01-26

**Authors:** Afsane Radmand, Melissa P. Lokugamage, Hyejin Kim, Curtis Dobrowolski, Ryan Zenhausern, David Loughrey, Sebastian G. Huayamares, Marine Z. C. Hatit, Huanzhen Ni, Ada Del Cid, Alejandro J. Da Silva Sanchez, Kalina Paunovska, Elisa Schrader Echeverri, Aram Shajii, Hannah Peck, Philip J. Santangelo, James E. Dahlman

**Affiliations:** † Petit Institute for Bioengineering and Biosciences, Georgia Institute of Technology, Atlanta, Georgia 30332, United States; ‡ Department of Chemical Engineering, Georgia Institute of Technology, Atlanta, Georgia 30332, United States; § Wallace H. Coulter Department of Biomedical Engineering, Georgia Institute of Technology and Emory University School of Medicine, Atlanta, Georgia 30332, United States

**Keywords:** DNA barcode, LNP, lipid nanoparticle, mRNA, single-cell RNA sequencing, scRNA-seq

## Abstract

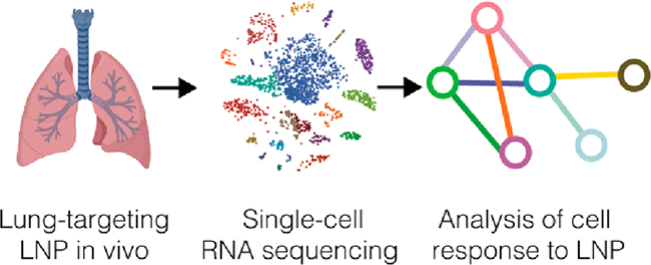

Lipid nanoparticles (LNPs) have delivered RNA to hepatocytes in patients, underscoring the potential impact of nonliver delivery. Scientists can shift LNP tropism to the lung by adding cationic helper lipids; however, the biological response to these LNPs remains understudied. To evaluate the hypothesis that charged LNPs lead to differential cellular responses, we quantified how 137 LNPs delivered mRNA to 19 cell types *in vivo*. Consistent with previous studies, we observed helper lipid-dependent tropism. After identifying and individually characterizing three LNPs that targeted different tissues, we studied the *in vivo* transcriptomic response to these using single-cell RNA sequencing. Out of 835 potential pathways, 27 were upregulated in the lung, and of these 27, 19 were related to either RNA or protein metabolism. These data suggest that endogenous cellular RNA and protein machinery affects mRNA delivery to the lung *in vivo*.

Lipid nanoparticles (LNPs) are clinically relevant delivery systems that have delivered mRNA-based COVID vaccines after intramuscular administration,^1,2^ mRNA and single-guide RNA^3,4^ to hepatocytes after intravenous administration, and siRNA to hepatocytes after intravenous administration.^5^ These data justify the exploration of LNPs that target nonliver tissues.^6^ Scientists use three approaches to increase nonliver mRNA delivery. One is to pretreat animals with drugs that block the liver from taking up LNPs^7^ or reduce the activity of mRNA once it reaches hepatocytes.^8,9^ A second is to add active targeting ligands to nanoparticles.^10^ For example, mRNA-loaded LNPs were targeted to Ly6c^+^ leukocytes utilizing antibodies that were anchored into the lipid membrane.^11^ In another example, plasmalemma vesicle-associated protein-targeting LNPs were covalently conjugated with antibodies, which increased lung protein expression.^12^ A third approach is to change the chemical composition of the LNP. Clinical LNPs have consisted of four components: an ionizable or cationic lipid, a poly(ethylene glycol) (PEG)-lipid, a cholesterol, and a helper lipid. For example, when a cationic helper lipid was added to a liver-targeting LNP, it was redirected to lung endothelial cells.^13^ In another example, RNA-lipoplexes were targeted to lymphoid-resident dendritic cells by titrating negative charge.^14^ Finally, a fifth charged molecule was added to the LNP, improving tropism to the spleen and lung.^15^ Notably, authors subsequently reported that these LNPs can bind different serum proteins, providing an insight into the biology of delivery.^16^ These studies led us to hypothesize that changing the helper lipid could affect the internal cellular response to LNPs *in vivo*.

We first confirmed helper lipid-dependent tropism. We used a functional *in vivo* mRNA and DNA barcode system^17−20^ to evaluate how 18 helper lipids affected tropism to 19 cell types *in vivo.* We microfluidically mixed^21^ nanoparticle components with nucleic acids at a lipid:nucleic acid mass ratio of 10:1 (Figure 1a). To isolate the effect of the helper lipid, the other three LNP components were previously validated; we used the ionizable lipid cKK-E12, a cholesterol, and two PEG-lipids. As a control to ensure delivery changes were not driven by a specific ratio of the four components, we formulated LNPs with four molar ratios (Figures 1b and S1). Each of the 18 helper lipids was thus formulated eight times, creating 144 chemically distinct LNPs (Figure S2).

**Figure 1:**
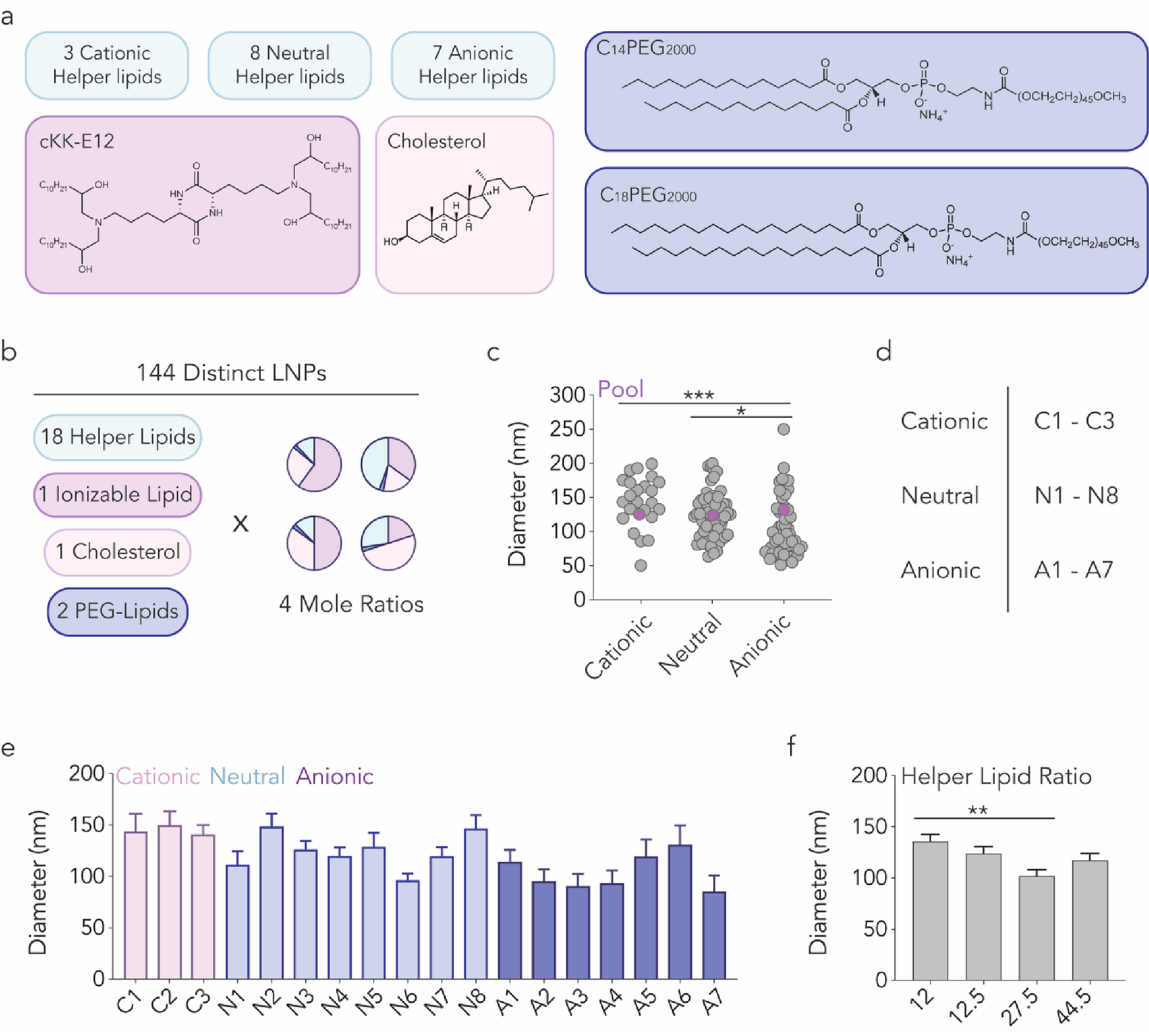
Characterizing how helper lipid composition influences LNP formation. (a, b) 144 chemically distinct LNPs were created by varying 18 helper lipids as well as the molar ratios of the LNPs’ four components. (c) Hydrodynamic diameter of all individual LNPs (gray) as well as the pool of LNPs that were mixed together (purple). The diameter of the pool was within the range of the LNPs composing that pool. *P = 0.0127, ***P = 0.0001, one-way ANOVA. (d, e) Diameter as a function of helper lipid. Average ± SEM. (f) Diameter as a function of helper lipid mole ratio. **P = 0.0034, one-way ANOVA, average ± SEM.

The 18 helper lipids varied in two traits. The first was charge: eight helper lipids were neutrally charged, seven were anionic, and three were cationic. Second, we varied helper lipid headgroup molecular weight and lipid tail saturation^22,23^ (Figure S2). After formulating the 144 LNPs, we investigated whether helper lipid composition influenced LNP hydrodynamic diameter and polydispersity using dynamic light scattering (DLS). We observed a relationship between helper lipid charge and LNP hydrodynamic diameter: LNPs with anionic helper lipids tended to form smaller particles (Figure 1c). We then quantified LNP hydrodynamic diameter as a function of the 18 helper lipids (Figures 1d and S2) but found no statistical relationship between helper lipids and size (Figure 1e). By measuring LNP hydrodynamic diameter as a function of helper lipid molar ratio, we found that LNPs with 27.5% of helper lipid formed smaller particles compared to those with 12% (Figure 1f). These results led us to conclude that stable four-component LNPs could be created using distinct helper lipids.

We then investigated whether helper lipid structure influenced mRNA delivery *in vivo*. We utilized Fast Identification of Nanoparticle Delivery (FIND) to quantify functional mRNA delivery (i.e., mRNA translated into protein) mediated by many LNPs in a single animal^17−19^ (Figure 2a). In this system, LNP-1, with chemical structure 1, is formulated to carry Cre mRNA and DNA barcode 1; LNP-N, with chemical structure N, is formulated to carry Cre mRNA and DNA barcode N. LNPs with a hydrodynamic diameter less than 200 nm and a monodisperse DLS spectrum are pooled and administered to Ai14 mice.^24^ Cells in which Cre mRNA has been translated into functional Cre protein, which edits the genome by excising the Lox-Stop construct from genomic DNA, become tdTomato^+^. tdTomato^+^ cells are subsequently sequenced to identify barcodes in functionally transfected cells. Thus, functional mRNA delivery is quantified as the percentage of tdTomato^+^ cells, and the contribution of each LNP is quantified by sequencing as normalized delivery^25−27^ (Figure S3a).

**Figure 2:**
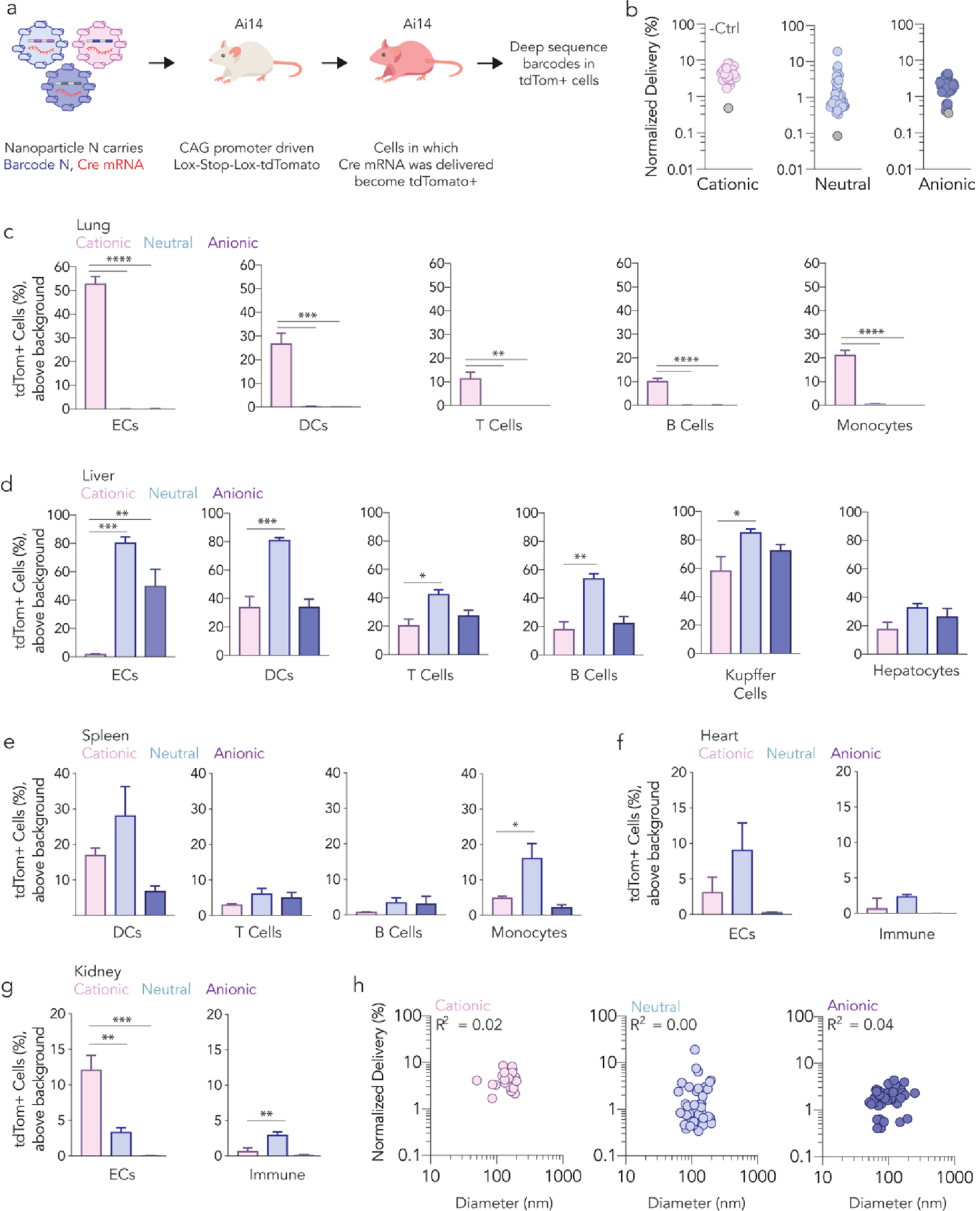
Helper lipid charge influences LNP *in vivo* tropism at the cellular level. (a) LNPs were formulated to carry a DNA barcode and Cre mRNA; 137 LNPs were pooled into their respective libraries and were then administered to Ai14 mice. After 4 days, tdTomato signal was quantified and tdTomato^+^ cells were isolated. Next-generation sequencing identified LNPs that functionally transfected cells *in vivo*. (b) Normalized delivery of all LNPs across all cell types in each library. The control, unencapsulated barcode, delivered less efficiently than barcodes delivered by LNPs. tdTomato^+^ signal in (c) lung, (d) liver, (e) spleen, (f) heart, and (g) kidney cells. These data suggest preferential delivery to lung cell types by the cationic LNPs; no delivery was observed by the neutral and anionic LNP pools to lung cell types. ****P < 0.0001, ***P < 0.0006, **P < 0.01, *P < 0.03, one-way ANOVA, average ± SEM. (h) Normalized delivery as a function of hydrodynamic diameter.

We performed three experiments, creating one library for each lipid charge. Of the 24 cationic LNPs we formulated, 23 met our 200 nm and single DLS peak selection criteria. These LNPs were pooled and intravenously injected into four mice at a total dose of 1.0 mg/kg nucleic acid (i.e., 0.043 mg/kg/LNP, on average). We formulated 64 LNPs with a neutral helper lipid; 59 met pooling criteria and were administered at a total dose of 1.0 mg/kg nucleic acid. Finally, we formulated 56 LNPs with an anionic helper lipid, of which 55 met pooling criteria and were administered to mice at a total dose of 1.0 mg/kg nucleic acid. Thus, across the three screens, we formulated 144 LNPs and injected 137 LNPs. Four days later, we sacrificed mice and quantified the percentage of tdTomato^+^ cells for 19 cell types, using phosphate-buffered saline (PBS)-treated mice as tdTomato gating controls (Figure S3b). As a sequencing control, we included a DNA barcode that was not encapsulated in an LNP. This barcode should be delivered into cells less efficiently than barcodes carried in LNPs. As expected, unencapsulated barcodes were delivered inefficiently (Figures 2b and S4). Relative to PBS-treated mice, mice treated with LNPs did not lose weight (Figure S5).

We then quantified mRNA delivery as a function of helper lipid charge in five lung cell types (Figure 2c). Lung cells in mice treated with cationic LNPs became tdTomato^+^; lung cells in mice treated with neutral or anionic LNPs did not (Figure 2c). In cationic LNP-treated mice, we observed over 50% tdTomato^+^ lung endothelial cells (ECs). Between 10% and 25% of the lung dendritic cells (DCs), T cells, B cells, and monocytes were tdTomato^+^, suggesting that the LNPs first targeted the endothelial cells, which are accessible from the bloodstream, and then targeted less accessible cells. In contrast, LNPs containing neutral or anionic helper lipids did not lead to measurable lung mRNA delivery but did lead to liver delivery (Figure 2d). In the spleen, heart, and kidney, we observed delivery, albeit at lower levels (Figure 2e–g). These data suggest that cationic helper lipid charge within four-component LNPs can increase the lung:liver delivery ratio. We observed no relationship between LNP hydrodynamic diameter and delivery (Figure 2h).

We used the barcoding readout to identify relationships between lipid structure and *in vivo* delivery (Figure S6). First, we explored whether helper lipid molar ratio affected LNP normalized delivery. In the neutral screen, LNPs with 27.5% of helper lipid had a higher normalized delivery compared to those with 12% and 44.5%. We did not observe a similar relationship in the cationic and anionic screens (Figure S7). Second, we calculated the fold change enrichment of each helper lipid in the lung, liver, and spleen. Enrichment^28^ determines how often a specific chemical property appears in particles that performed in the top 10% and bottom 10%, relative to chance; fold enrichment is calculated by subtracting enrichment in the bottom 10% from enrichment in the top 10% (Figure S8a). Enrichment analyses (Figures S8–S11) suggested four relationships between lipid structure and delivery. First, 18:0 dimethyldioctadecylammonium (DDAB) was the most enriched cationic helper lipid in all cell types, across all tissues (Figure S8b–d). Second, 18:1 1,2-dioleoyl-sn-glycero-3-phosphate (PA) was most enriched in all liver and spleen cell types in the anionic library (Figure S9a–c). Third, 1,2-distearoyl-sn-glycero-3-phosphocholine (DSPC) was positively enriched across liver and spleen cell types (Figure S10a–c) and was found in the best-performing LNP (highest normalized delivery) from the neutral screen (Figure S6a). Fourth, helper lipids with smaller headgroups tended to promote LNP delivery more than helper lipids with larger headgroups; we compared the enrichment of four neutral helper lipids with the same tail but different headgroups. Headgroups with lower molecular weights, ammonium and methylamine, were positively enriched, whereas headgroups with higher molecular weights, dimethylamine and trimethylamine, were negatively enriched (Figure S11a–c). These data suggest that lipid self-assembly and packing, which are influenced by headgroup size,^29^ influence *in vivo* delivery.

Predictions made by high-throughput *in vivo* screens should be confirmed using individual LNPs. Thus, we characterized a lead LNP from each library. We named the lead LNP from the cationic screen Cat-LNP (Figure 3a); the winners from the neutral and anionic screens were similarly named Neu-LNP and An-LNP (Figure S15a,c). All three lead LNPs formed monodisperse structures characterized by DLS and transmission electron microscopy (Figure S12).

**Figure 3:**
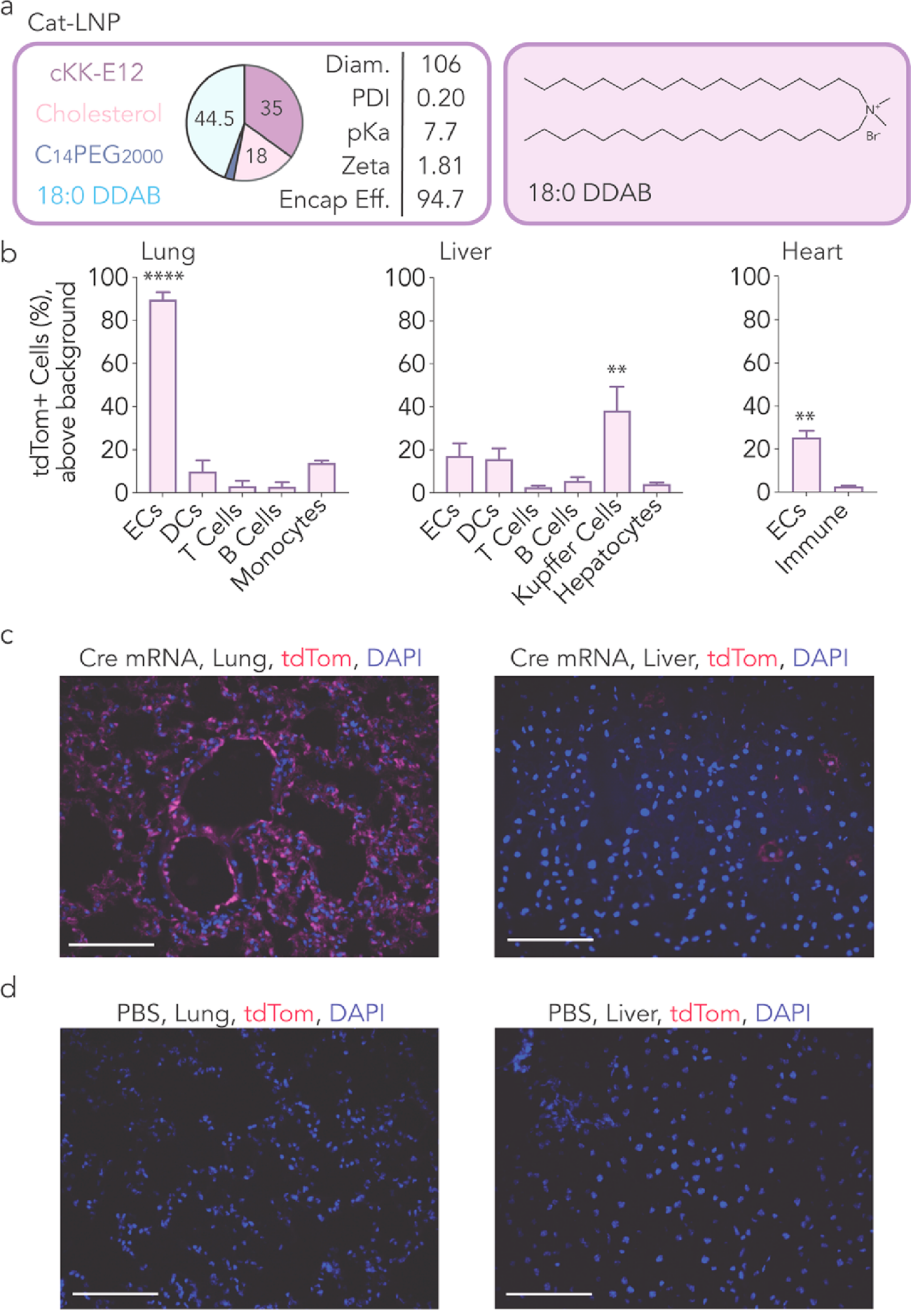
Top LNP from cationic screen delivers mRNA potently to lung ECs. (a) An LNP was identified from the cationic screen and named Cat-LNP. (b) The Cat-LNP delivered to lungs more than liver. A cationic helper lipid, 18:0 DDAB and cKK-E12, cholesterol, and C14PEG2000 were used to formulate the Cat-LNP. The Cat-LNP was administered to mice intravenously at a dose of 1.3 mg/kg. Four days later, tdTomato^+^ cells were quantified. We found that the Cat-LNP delivered Cre mRNA significantly to lung ECs, heart ECs, and liver Kupffer cells. ****P < 0.0001, **P < 0.006, two-way ANOVA, average ± SEM. (c, d) Lung and liver tissues were imaged, and tdTomato signal was quantified. Scale bars represent 100 μm. Diam.: Diameter in nm; Zeta: Zeta potential in mV; Encap Eff.: Encapsulation efficiency in %.

We intravenously injected Cat-LNP carrying Cre mRNA into Ai14 mice at a dose of 1.3 mg/kg; 4 days later, we quantified the percentage of tdTomato^+^ cells in the lungs, liver, spleen, kidney, and heart. Cat-LNP showed over 90% mRNA encapsulation efficiency and positive zeta potential (Figure 3a). Consistent with the screen, flow cytometry revealed that Cat-LNP delivered mRNA to multiple cell types in the lung (Figures 3b and S13a) more than the liver. This was confirmed by tdTomato imaging (Figure 3c,d). Cat-LNP also delivered mRNA to kidney and heart endothelial cells (Figures 3b and S13a). We then intravenously injected Cat-LNP at a dose of 0.3 mg/kg, comparing it to a previously reported lung-targeting LNP^13^ (Figure S14a). Compared to the control LNP, Cat-LNP significantly enhanced functional mRNA delivery to heart and kidney endothelial cells (Figure S14b–f).

Next, we confirmed the activity of Neu-LNP (Figure S15a). As predicted by the screen, Neu-LNP preferentially delivered to liver cell types (Figure S15b). We also noted delivery to splenic DCs and kidney ECs and monocytes (Figure S13b). Finally, we tested An-LNP (Figure S15c). We observed preferential delivery to liver cell types (Figures S15d and S13c) with the highest delivery to endothelial cells (Figure S15d). To complement these tdTomato readouts, which quantify functional delivery, we analyzed Cat-LNP, Neu-LNP, and An-LNP using QUANT, a PCR-based approach for measuring LNP biodistribution.^27^ Consistent with our Cre-mediated functional mRNA delivery readouts, Cat-LNP biodistribution was higher in the lung compared to Neu-LNP and An-LNP (Figure S16a–c).

To control for potential mRNA sequence-specific effects, we formulated Cat-LNP, Neu-LNP, and An-LNP with mRNA encoding anchored vHH (aVHH)^30−32^ and intravenously injected them into BL/6 mice at a dose of 1.3 mg/kg (Figure S17a,b). Cat-LNP again delivered aVHH mRNA to all lung cell types, whereas Neu-LNP and An-LNP did not (Figure S17d). We then quantified serum cytokines 6 h after LNP administration, using lipopolysaccharide (LPS) as a positive control. Compared to PBS-injected mice, we observed cytokine elevation at 6 h (Figure S18). Early cytokine increases are observed in clinical LNPs and typically resolve by 24 h.^33^ We also monitored weight loss and found no change relative to PBS-treated mice (Figure S19).

Given that Cat-LNP, Neu-LNP, and An-LNP targeted different tissues, we reasoned that they could be used to study the transcriptional response to LNPs in the lung. We therefore utilized single-cell RNA sequencing (scRNA-seq) to understand how lung cells responded after mice were injected with LNPs. We intravenously injected BL/6 mice with Cat-LNP, Neu-LNP, and An-LNP carrying aVHH mRNA at a dose of 1.0 mg/kg. One hour later, we sacrificed the mice and isolated single cells from the lungs. We processed the resulting data using Seurat^34^ and analyzed it through BBrowser from BioTuring.^35^ Unsupervised clustering partitioned the lung cells into 27 clusters (Figures 4a and S20a). These cell populations were consistent with previous scRNA-seq data sets from the lung^36,37^ and did not change in mice treated with LNPs relative to mice treated with PBS (Figure 4a).

**Figure 4:**
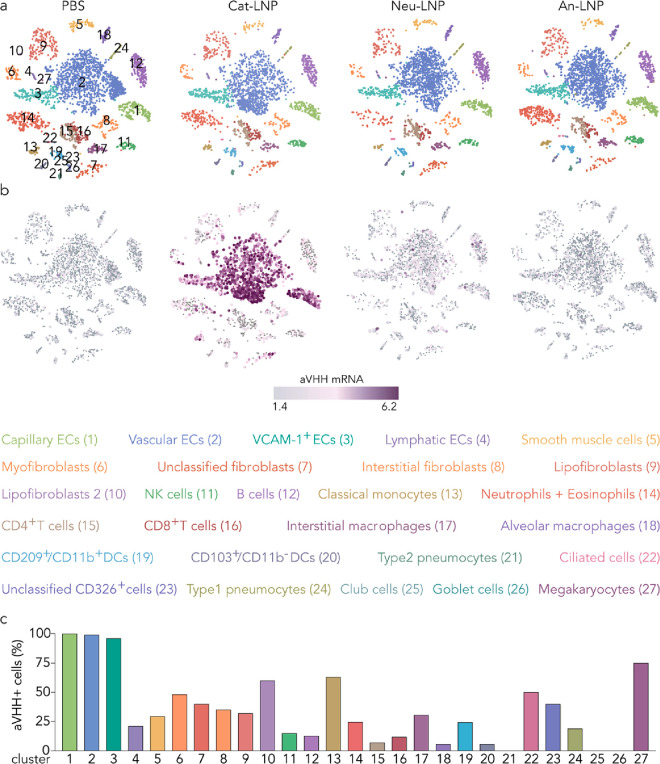
Top LNP from cationic screen (Cat-LNP) potently delivers aVHH mRNA to lung ECs. (a) Unsupervised clustering partitioned the lung into clusters of endothelial cells, fibroblasts, epithelial cells, and immune cell subtypes. (b) Cat-LNP delivered aVHH mRNA to subtypes of lung ECs more than the control group (Neu-LNP, An-LNP, and PBS) at the 1 h time point. (c) The percentage of aVHH^+^ cells for each of the 27 clusters.

To analyze the delivery in all 27 lung cell types, we first used BBrowser to visualize the clusters using T-distributed stochastic neighbor embedding (t-SNE)^38^ (Figures 4a and S20a). We then created an aVHH pseudogene and overlaid it on the t-SNE, thereby analyzing the amount of aVHH mRNA delivered with single-cell resolution. Consistent with flow cytometry results, we measured more aVHH reads in lung endothelial cells than other lung cells in mice treated with Cat-LNP (Figures 4b and S20b,c). The highest aVHH mRNA delivery was in capillary ECs (Ednrb^+^, Tmem100^+^, Vwf^–^), vascular ECs (Tmem100^+^, Vwf^–^, Ednrb^–^), VCAM-1^+^ ECs (Vwf^+^, Tmem100^+^, Plac8^+^), and interestingly, lymphatic ECs (Mmrn1^+^). Cat-LNP also delivered aVHH mRNA to lung fibroblasts (Col1a2^+^), interstitial fibroblasts (Col1a2^+^, Dcn^+^, Inmt^+^), lipofibroblasts (Inmt^+^, Col1a2^+^ (low), Dcn^–^), myofibroblasts (Col1a2^+^, Wif1^+^, Fgf18^+^, Aspn^+^), and smooth muscle cells (Col1a2^+^, Acta2^+^). We observed delivery to lung immune cells including classical monocytes (Plac8^+^, Ly6c2^+^), neutrophils (Ngp^+^) and eosinophils (Cxcr2^+^), B cells (Cd79b^+^), NK cells (Gzma^+^), CD209^+^ CD11b^+^ DCs (CD209a^+^, Ccl17^+^, Ear2^+^), CD8^+^ T cells (Trbc2^+^, Ly6c2^+^, Cd8b1^+^), and interstitial macrophages (C1qb^+^, Prg4^–^). Finally, we measured delivery to ciliated cells (CD326^+^, Foxj1^+^), CD326^+^ cells, type 1 pneumocytes (Rtkn2^+^), and megakaryocytes (Ppbp^+^) (Figure 4c). Consistent with earlier results, Neu-LNP and An-LNP delivered less mRNA to the lung than Cat-LNP (Figure 4b).

After confirming delivery to lung cells in mice treated with Cat-LNP, we quantified transcriptomic changes in lung endothelial cells. We used a differential expression tool in BBrowser to analyze genes that exhibited differential expression, comparing cells from mice treated with Cat-LNP to cells from mice treated with PBS, Neu-LNP, or An-LNP. We used volcano plots to visualize the data and included the genes with *P*-value < 0.05 (Figure 5a). We found that 243 genes were significantly upregulated and 145, downregulated with Cat-LNP when compared to PBS; 283 were upregulated and 101, downregulated when compared to Neu-LNP; 213 were upregulated and 114, downregulated when compared to An-LNP.

**Figure 5:**
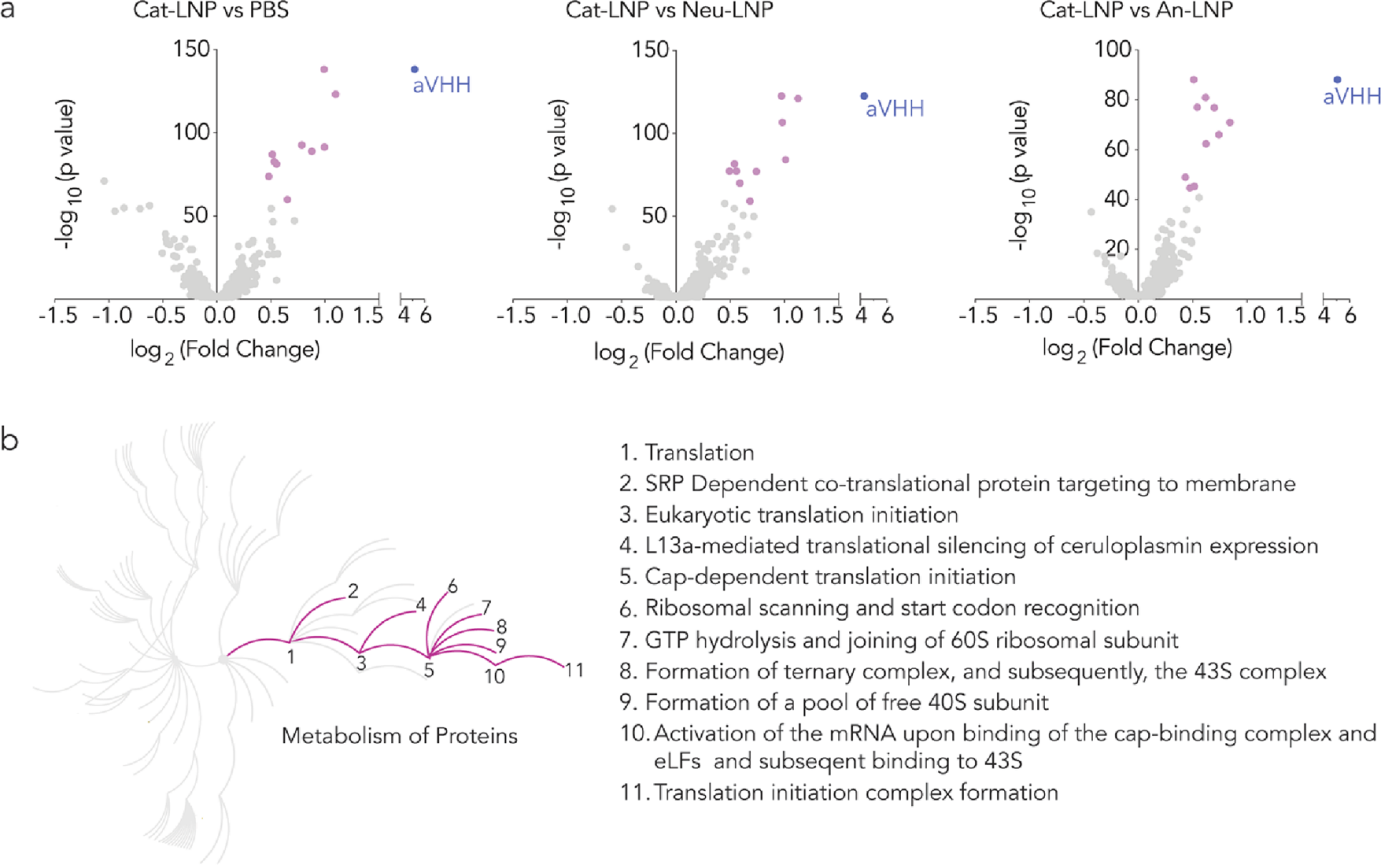
Cat-LNP significantly changes the transcriptomic profile of lung ECs and leads to upregulation of pathways related to metabolism of proteins (R-MMU-392499). (a) Differentially expressed genes in lung ECs in response to Cat-LNP when compared to control groups, i.e., PBS, Neu-LNP, and An-LNP. Purple dots represent the top 10 upregulated genes, *P*-value < 0.05. (b) Reactome pathway analysis with significantly upregulated pathways highlighted and listed, *P*-value < 0.001.

Notably, of the top 10 upregulated genes when Cat-LNP was compared to PBS (Figure 5a, purple), eight (Nfkbia, Zfp36, Icam1, Rhob, Tnfaip3, Lmna, Myadm, and Fosb) were also upregulated when Cat-LNP was compared to Neu-LNP or An-LNP (Figure S21). This data led us to conclude that Neu-LNP and An-LNP did not substantially change the biological response in lung ECs, which is consistent with lower delivery to the lung.

The consistent transcriptomic response in Cat-LNP-treated mice, relative to the PBS-, Neu-LNP-, and An-LNP-treated mice, led us to conclude that the gene expression profile was valid. We therefore used the Reactome pathway database^39^ to understand the cellular processes implicated by these changes. Of 835 potential pathways, 27 were enriched in mice treated with Cat-LNP (*P*-value < 0.001, Figure S22). Notably, 19 of the 27 were related to the metabolism of either RNA or protein. Specifically, 11 of the 27 pathways were related to protein metabolism (R-MMU-392499, Figure 5b), including translation initiation (Reactome pathway ID: R-MMU-72649, R-MMU-72737, R-MMU-72613) and ribosomal assemblies required for subsequent translation (R-MMU-72702, R-MMU-72662, R-MMU-72689, R-MMU-72695, R-MMU-72706). Similarly, eight were related to RNA metabolism (R-MMU-8953854), including rRNA processing (R-MMU-72312, R-MMU-6791226, R-MMU-8868773) and regulation of mRNA stability (R-MMU-450531, R-MMU-450408). These data suggest that lung endothelial cells respond to LNPs carrying mRNA in part by upregulating genes related to the manufacture and processing of RNA and subsequently produced proteins.

LNP-mRNA therapies will require a biological understanding of the way cells respond to these delivery systems. Here we found consistent evidence that a four-component LNP with a cationic helper lipid delivered mRNA to the lung more efficiently than LNPs with a neutral or anionic helper lipid. Interestingly, delivery within the lung, although highest in endothelial cells accessible from the blood, extended to other cell types, including subtypes that are difficult to assay using traditional techniques. By combining aVHH delivery with scRNA-seq, thereby analyzing delivery in transcriptionally defined cells, we found evidence of LNP delivery to fibroblasts, a suite of lung immune cells, lymphatic endothelial cells, and even epithelial cells. Notably, the efficiency of delivery across cell types was many-fold. Although additional work is required, this suggests that Cat-LNP may first saturate endothelial cells and then target additional cell types. In turn, this leads to two interesting questions. First, is this same effect observed in other tissues with continuous vasculature? Second, can LNPs be designed with tropism that is more evenly distributed across lung cell types? These data justify studies using scRNA-seq to characterize *in vivo* delivery as well as studies to identify the physical mechanism by which these LNPs access lung parenchyma.

When we analyzed the transcriptomic data, two facts struck us. First, only 27 of the 835 pathways were affected by LNP delivery. Second, 19 of the 27 were related to RNA and protein metabolism. These data suggest that cells respond to LNP-mRNA drugs in large part by changing the way mRNA and subsequent proteins are processed. We noted that several steps related to early translation were impacted, including translation initiation, complex formation, and ribosomal scanning and start codon recognition. We were also especially interested in the role of cap-dependent translation initiation since synthetic mRNA can be rationally engineered for mRNA therapies.^40^ The data provide a very early line of evidence that cap-dependent translation can be measured and, therefore, optimized *in vivo* after LNP treatment. The data also imply, but do not prove, that manipulating these pathways using small molecules could impact LNP delivery. We therefore envision future studies pretreating cells to “prime” them for LNP delivery. One limitation of this work is that we did not knock out genes *in vivo* and then observe changes in delivery, which is the gold standard^41,42^ required to confirm whether a specific gene affects delivery.

It is important to acknowledge other key limitations of this work. First, the experiments were performed in mice. The RNA and protein metabolism response will therefore need to be observed in larger animals.^31^ Second, the cellular response could change with the delivery vehicle. We envision repeating these studies with other nanomaterials. Third, the study does not include a systematic comparison of the microenvironmental response in different tissues; we hope that future work will address this question. Despite these limitations, we believe these data provide insights into the early cellular responses of LNP-mRNA drugs in a tissue that could be clinically relevant within the next few years.

## Supporting Information Available

Materials and methods, LNPs molar ratios and helper lipid structures, example schematic of normalized delivery, cell type-specific markers, normalized delivery of LNPs, mice weight measures, enrichment analysis, TEM images of LNPs, tdTomato and aVHH protein expression quantification using FACS, biodistribution profiling, cytokine analysis, t-SNE plots, cell markers and aVHH mRNA expression profiling, aVHH mRNA expression in new cell types, volcano plots, eeactome pathways, and gating strategy for FACS (PDF)
