# Supplementary material for: The Transcriptional Response to Lung-Targeting Lipid Nanoparticles in Vivo
Source: Nano Lett. 2023 Jan 26;23(3):993–1002. doi: 10.1021/acs.nanolett.2c04479 (PMC9912332; doi:10.1021/acs.nanolett.2c04479)
Supplement: Supplementary file 1 — nl2c04479_si_001.pdf [file nl2c04479_si_001.pdf]

**Supporting Information**

# The transcriptional response to lung-targeting lipid nanoparticles *in vivo*

Afsane Radmand[1,2], Melissa P. Lokugamage[3], Hyejin Kim[3], Curtis Dobrowolski[3], Ryan Zenhausern[3], David Loughrey[3], Sebastian G. Huayamares[3], Marine Z. C. Hatit[3], Huanzhen Ni[3], Ada Del Cid[3], Alejandro J. Da Silva Sanchez[1,2], Kalina Paunovska[3], Elisa Schrader Echeverri[3], Aram Shajii[3], Hannah Peck[3], Philip J. Santangelo[3], James E. Dahlman*[3]

[1]Petit Institute for Bioengineering and Biosciences, Georgia Institute of Technology, Atlanta, GA, 30332, USA

[2]Department of Chemical Engineering, Georgia Institute of Technology, Atlanta, GA, 30332, USA

[3]Wallace H. Coulter Department of Biomedical Engineering, Georgia Institute of Technology and Emory University School of Medicine, Atlanta, GA, 30332, USA

*Correspondence: james.dahlman@emory.edu

## Materials and Methods

### Cre mRNA synthesis

mRNA was synthesized as described previously[39]. Briefly, the mRNA sequence was ordered as a DNA gBlock from Integrated DNA Technologies (IDT) containing a 5' UTR with Kozak sequence, a 3' UTR derived from the mouse alpha-globin sequence, and extensions to allow for Gibson assembly. The sequence was human codon-optimized using the IDT website. The sequences of the GPI anchor and nano-luciferase have been previously stated[39]. The gBlock was then cloned into a PCR-amplified pMA7 vector through Gibson assembly using NEB Builder with 3 molar excess of insert. All reaction transcripts were 0.8% agarose gel purified prior to assembly reaction. Subsequent plasmids from each colony were Sanger sequenced to ensure desired sequence fidelity. Plasmids were linearized with NotI-HF (New England BioLabs, NEB) overnight at 37 °C. Linearized templates were purified by ammonium acetate (Thermo Fisher Scientific) precipitation before being rehydrated with nuclease-free water. In vitro transcription (IVT) was performed overnight at 37 °C using the HiScribe T7 kit (NEB) following the manufacturer's instructions (N1-methyl-pseudouridine modified). RNA product was treated with DNase I (Aldevron) for 30 min to remove template and purified using lithium chloride precipitation (Thermo Fisher Scientific). RNA was heat-denatured at 65 °C for 10 min before being capped with a Cap1 structure using guanylyl transferase (Aldevron) and 2'-O-methyltransferase (Aldevron). Transcripts were then polyadenylated enzymatically (Aldevron). mRNA was then purified by lithium chloride precipitation, treated with alkaline phosphatase (NEB), and purified again. Concentrations were measured using a NanoDrop. mRNA stock concentrations were 3–5 mg/mL. Purified RNA products were analyzed by gel electrophoresis to ensure purity.

**aVHH mRNA synthesis**

mRNA was synthesized as previously described[29-31]. In summary, the GPI- anchored VHH sequence was ordered as a DNA gBlock from IDT containing a 3’ UTR derived from the mouse alpha-globin sequence, 5’ UTR with Kozak sequence, and extensions to allow for Gibson assembly. The sequence was human codon-optimized using the IDT website. The gBlock was then cloned into a PCR amplified pMA7 vector through Gibson assembly using NEB Builder with 3 molar excess of insert. Gibson assembly reaction transcripts were 0.8% agarose gel purified prior to assembly reaction. Subsequent plasmids from each colony were Sanger sequenced to ensure sequence identity. Plasmids were digested into a linear template using NotI-HF (New England BioLabs) overnight at 37°C. Linearized templates were purified by ammonium acetate (Thermo Fisher Scientific) precipitation before being resuspended with nuclease-free water. IVT was performed overnight at 37 °C using the HiScribe T7 kit (NEB) following the manufacturer’s instructions (full replacement of uracil with N1-methyl-pseudouridine). RNA product was treated with DNase I (Aldevron) for 30 min to remove template and purified using lithium chloride precipitation (Thermo Fisher Scientific). RNA transcripts were heat denatured at 65 °C for 10 min before being capped with a Cap1 structure using guanylyl transferase (Aldevron) and 2’-O-methyltransferase (Aldevron). Transcripts were then polyadenylated enzymatically (Aldevron). mRNA was then purified by lithium chloride precipitation, treated with alkaline phosphatase (NEB), and purified a final time. NanoDrop was used to measure RNA concentration; mRNA stock concentrations were between 2 and 4 mg/mL and the mRNA stock was stored at -80°C. Purified RNA products were analyzed by gel electrophoresis to ensure purity.

**Nanoparticle Formulation**

Nanoparticles were formulated using a microfluidic device as previously described[40]. Cre mRNA and DNA barcodes were diluted in 10 mM citrate buffer (Teknova). DNA barcodes were purchased from IDT. cKK-E12 was purchased from Organix Inc. (O-8744). PEGs, cholesterol, and helper lipids were diluted in 100% ethanol and purchased from Avanti Lipids. Citrate and ethanol phases were combined in a microfluidic device by syringes (Hamilton Company) at a flow rate of 3:1.

**Nanoparticle Characterization**

The diameter and polydispersity of the LNPs were measured using dynamic light scattering (DLS) (DynaPro Plate Reader II, Wyatt). LNPs were diluted in sterile 1X PBS and analyzed. To avoid using unstable LNPs, and to enable sterile purification using a 0.22 µm filter, LNPs were included only if they met 3 criteria: diameter >50 nm, diameter <200 nm, and correlation function with 1 inflection point. For screens, particles that met these criteria were pooled into respective groups. Particles were dialyzed in Slide-A-Lyzer G2 20 kD dialysis cassettes (Thermo Scientific) and then dialyzed in Float-A-Lyzer G2 100 kD dialysis cassettes (Millipore Sigma). The nanoparticle concentration was determined using NanoDrop (Thermo Scientific).

**Encapsulation efficiency**

Encapsulation was measured according to Precision NanoSystems RiboGreen assay protocol. In duplicates, 50 µL of 6 ng/µL LNP was added to 50 µL of 1X TE (Thermo Fisher) or 50 µL of

solution containing a 1:50 dilution of Triton X-100 (Sigma Aldrich). Following 10 minutes of incubation at 37 °C, 100 µL of 1:100 of RiboGreen reagent (Thermo Fisher) was added to each well. The fluorescence was quantified using a plate reader (BioTek Synergy H4 Hybrid) at an excitation wavelength of 485 nm and an emission wavelength of 528 nm.

**Zeta Potential (ZP)**
The zeta potential of LNPs was measured using a Malvern Zetasizer Nano Z. Eight hundred microliters of the particles were loaded into a Malvern disposable folded capillary cell and the following settings were executed: material refractive index of 1.4, absorbance of 0.01, dispersant viscosity of 0.882cp, refractive index of 1.33, and dielectric constant of 79.

**TEM imaging**
The lipid nanoparticles were received in an aqueous solution. Ten microliters of solution was dropped onto a copper TEM grid covered with Formvar support film and stabilizing carbon. The excess solution was blotted with filter paper and the negative stain was added. The particles were stained for 1-2 minutes, using either 2% phosphotungstic acid or UranyLess (Electron Microscopy Sciences, Hatfield, PA) then blotted and allowed to air dry for several minutes before examination in the TEM (JEOL, Tokyo, Japan) at 100 kV.

**TNS Assay**
The pKa of Cat-LNP, Neu-LNP, and An-LNP was measured as previously described[40]. A stock solution of 10 mM HEPES (Sigma Aldrich), 10 mM MES (Sigma Aldrich), 10 mM sodium acetate (Sigma), and 140 mM sodium chloride (Sigma Aldrich) was prepared and pH adjusted with hydrogen chloride and sodium hydroxide to the following pH: 3, 4, 5, 6, 7, 8, 9, 10. Using four replicates for each pH, 140 µL pH-adjusted buffer was added to a 96-well plate, followed by adding 5 µL of 2-(p-toluidino)-naphthalene-6-sulfonic acid (60 µg/mL). Five microliters of LNP was added to each well. After 5 minutes of incubation at 300 rpm, fluorescence absorbance was measured using excitation wavelengths of 325 nm and emission wavelength of 435 nm using a plate reader (BioTek Synergy H4 Hybrids)

**Animal Experiments**
All animal experiments were performed in accordance with the Georgia Institute of Technology's IACUC. All animals were housed in the Georgia Institute of Technology Animal Facility. Ai14s were bred at the Georgia Institute of Technology Animal Facility. C57BL/6J (B6/000664) were purchased from Jackson Laboratories. In all experiments, we used N = 2-4 mice/group, unless otherwise noted.

**Cell Isolation & Staining**
Cells were isolated 24 or 96 hours after injection with LNPs, unless otherwise noted. Mice were perfused with 20 mL of 1X PBS through the right atrium. Tissues were finely cut, and then placed in a digestive enzyme solution with collagenase type I (Sigma Aldrich), collagenase XI (Sigma Aldrich) and hyaluronidase (Sigma Aldrich) at 37 ºC at 550 rpm for 45 minutes. The digestive enzyme for heart included collagenase IV. For the spleen, 1X PBS without digestive enzymes was used. Cell suspension was filtered through 70 µm mesh, and red blood cells were

lysed. Fc receptors were blocked (TruStain fcX™ anti-mouse CD16/32, BioLegend) to avoid non-specific binding. Then, cells were stained to identify specific cell populations and sorted using the BD FACS Fusion cell sorters in the Georgia Institute of Technology Cellular Analysis Core. The antibody clones used were anti-CD31 (390, BioLegend), anti-CD45 (30-F11, BioLegend), anti-CD68 (FA11, BioLegend), anti-CD11b (M1/70, BioLegend), anti-CD11c (N418, BioLegend), CD3 (17A2, BioLegend), CD19 (6D5, BioLegend), LIVE/DEAD™ Fixable Far-Red Dead Cell Stain (Invitrogen), and PE anti-mCD47 (miap301, BioLegend). Representative flow gates are listed in **Supplementary Fig. 23**. PBS-injected Ai14 mice were used to gate tdTomato-positive populations.

**PCR Amplification**

All samples were amplified and prepared for sequencing using a one-step PCR protocol as previously described[20]. More specifically, 1 µL of primers (5 µM for Final Reverse / Forward, 0.5 µM for Base Forward) were added to 5 µL of Kapa HiFi 2X master mix and 4 µL template DNA/water. When the PCR reaction did not produce clear bands, the primer concentrations, DNA template input, PCR temperature, and number of cycles were optimized for individual samples.

**Deep Sequencing**

Illumina deep sequencing was performed on an Illumina MiniSeq™. Primers were designed based on Nextera XT adapter sequences.

**Data Normalization**

Counts for each particle, per tissue, were normalized to the barcoded LNP mixture we injected into the mouse. This “input” DNA provided the DNA counts and was used to normalize DNA counts from the cells and tissues.

**Data Analysis & Statistics**

Sequencing results were processed using a custom Python-based tool to extract raw barcode counts for each tissue. These raw counts were then normalized with an R script prior to further analysis. Statistical analysis was done using GraphPad Prism 8. Data is plotted as mean ± standard error mean or mean ± standard deviation,

**Cytokine Analysis**

Six hours after LNP administration, blood was isolated from the heart. Serum was isolated and pooled (N=3) for each experimental condition, and cytokine levels were detected using the Mouse Cytokine Profiler Array Panel A (R&D Systems) following the manufacturer’s instructions. The final panels were documented using a LI-COR Odyssey CLx Far Infrared Imager and relative concentrations were determined using ImageJ software by determining pixel intensities.

**ddPCR**

The QX200 Droplet Digital PCR System (Bio-Rad) was used to analyze all ddPCR results.

ddPCR samples were prepared with 10 µL of ddPCR with ddPCR Supermix for Probes (Bio-Rad), 1 µL of primer and probe mix (solution of 10 µM target probe and 20 µM reverse/forward primers), 1 µL of template/TE buffer, and 8 µL of water. Once prepared, 20 µL of each reaction and 70 µL of Droplet Generation Oil for Probes (Bio-Rad) were loaded into DG8 Cartridges and covered with DG8 Gaskets. Using the QX200 Droplet Generator, water−oil emulsion droplets were created. Cycle conditions for PCR were as follows: 1 cycle of 95 °C for 10 min followed by 40 cycles of 94 °C for 30 s, 60 °C for 1 min, and 1 cycle of 95 °C for 10 min. For each biological replicate, three technical repetitions were completed. Unless stated otherwise, technical replicates were averaged. Technical replicates were only excluded if saturation was detected or there were inconsistent positive event amplitudes.

**Tissue Immunostaining & Microscopy**

Ai14 mice were treated with PBS or 1.3 mg/kg of Cre mRNA encapsulated in LNPs. Four days later, lung and liver tissues were dissected and fixed in 1 mL of 4% PFA for 24 hours. Cells were washed with 1X PBS. Tissues were cryoprotected in 30% sucrose for 48 hours at 4 °C. In tissue base molds, tissues were embedded in optimal cutting temperature compound (Tissue-Tek) and frozen. Blocks were stored at -80 °C. Tissues were sectioned through the Emory Winship Cancer Institute Cancer Tissue and Pathology Facility. Cells were stained with DAPI (Sigma). Images for a Zeiss Plan-Apo 20x 0.8 NA air objective. Images were captured and processed using Volocity software (PerkinElmer).

**Cell isolation for scRNA-seq**

One-hour post-injection, lungs were dissected from mice treated with Cat-LNP, Neu-LNP, An-LNP, and PBS. Lung cell suspensions were passed through 70 µm mesh and resuspended in RoboSep buffer (Stemcell Technologies) for further scRNA-seq processing.

**Single-cell library preparation**

Whole transcriptome analyses, using the BD Rhapsody Single-Cell Analysis System (BD, Biosciences), were performed on whole lung cells, following the manufacturer's protocol. Briefly, dead cells and red blood cells (RBCs) were depleted by using EasySep™ dead cell (Annexin V) and RBC (anti-TER119) removal kit (Stemcell Technologies) following the manufacturer's protocol. Cell viabilities and numbers were recorded for each sample and pooled at the same ratio. For a pooled sample, a BD Rhapsody cartridge was loaded with 40,000 cells. cDNA libraries were prepared using the BD Rhapsody Whole Transcriptome Analysis Amplification Kit following the BD Rhapsody System mRNA Whole Transcriptome Analysis (WTA) and Sample Tag Library Preparation protocol (BD Biosciences). The final libraries were quantified using a Qubit Fluorometer, and the size distribution was measured using Experion™ automated electrophoresis system (Bio-Rad).

**Processing of single-cell transcriptomics data**

The data were processed using zUMIs (v 2.9.7) for the RNA mapping and counting and Salmon Alevin (v1.5.2) for the cell hashes[41, 42]. All samples were mapped to GRCm39, and only exonic regions were counted. All output files were loaded into Seurat (v 4.0.4), and in summary, cells were log normalized to a scale factor of 10,000, then scaled using a linear transformation[33].

DoubletFinder (v3) was used to identify doublets as previously described[43]. This was followed by PCA dimensional reduction and t-SNE clustering and then exported using rBCS for further analysis in BBrowser2 (v2.9.23). Once in BBrowser2, the cell search tool was used to identify the cell types within each cluster, and gene expression profiles were compared within cell types of interest. Reactome pathway analysis was performed using ReactomeGSA package[38] in R based on Reactome database 82. The pathway expression levels are shown as z-score normalized values.

## Supplementary figures table of contents

Supplementary Figure 1 .......... 8
Supplementary Figure 2 .......... 11
Supplementary Figure 3 .......... 12
Supplementary Figure 4 .......... 13
Supplementary Figure 5 .......... 14
Supplementary Figure 6 .......... 17
Supplementary Figure 7 .......... 18
Supplementary Figure 8 .......... 19
Supplementary Figure 9 .......... 20
Supplementary Figure10 .......... 21
Supplementary Figure11 .......... 22
Supplementary Figure12 .......... 23
Supplementary Figure13 .......... 25
Supplementary Figure14 .......... 26
Supplementary Figure15 .......... 27
Supplementary Figure16 .......... 28
Supplementary Figure17 .......... 30
Supplementary Figure18 .......... 31
Supplementary Figure19 .......... 32
Supplementary Figure20 .......... 34
Supplementary Figure21 .......... 35
Supplementary Figure22 .......... 36
Supplementary Figure23 .......... 41

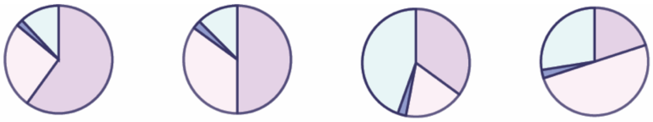

| Molar Ratio | | | | |
|---|---|---|---|---|
| Ionizable lipid | 60 | 50 | 35 | 20 |
| Cholesterol | 26 | 35 | 18 | 50 |
| PEG-Lipid | 2 | 2.5 | 2.5 | 2.5 |
| Helper Lipid | 12 | 12.5 | 44.5 | 27.5 |

**Supplementary Figure 1.** Molar ratios used in the LNP screens.

a

| Name | Charge | ID | Structure |
|---|---|---|---|
| DOTAP | Cationic | C1 | 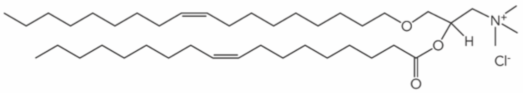 |
| DOTMA | Cationic | C2 | 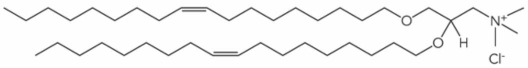 |
| 18:0 DDAB | Cationic | C3 | 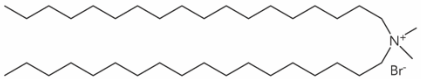 |

b

| Name | Charge | ID | Structure |
|---|---|---|---|
| 18:1 Lyso PE | Neutral | N1 | 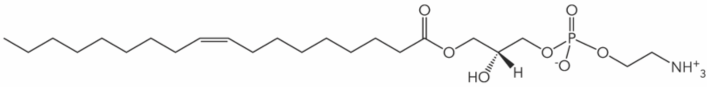 |
| 18:1 Lyso PC | Neutral | N2 | 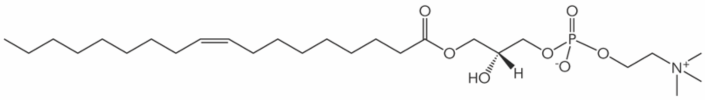 |
| DOPC | Neutral | N3 | 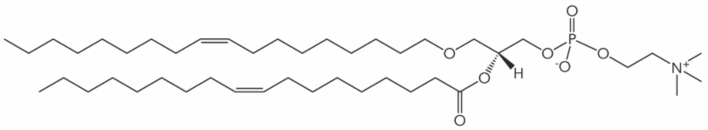 |
| DOPE | Neutral | N4 | 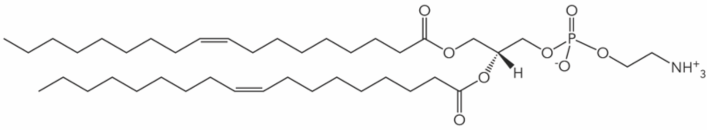 |
| 18:1 Monomethyl PE | Neutral | N5 | 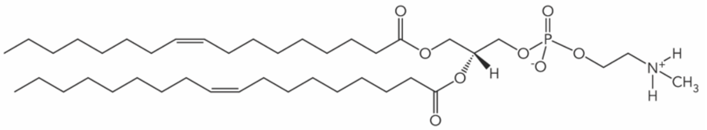 |
| 18:1 Dimethyl PE | Neutral | N6 | 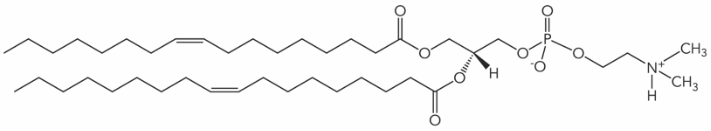 |
| 18:0 PC | Neutral | N7 | 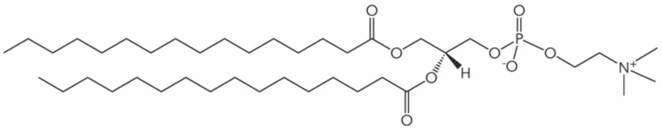 |
| 18:1 Caproylamine PE | Neutral | N8 | 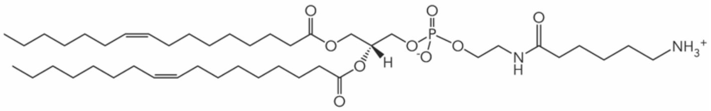 |

c

| Name | Charge | ID | Structure |
|---|---|---|---|
| 18:1 PA | Anionic | A1 | 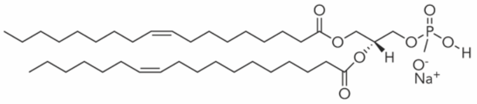 |
| 18:1 Phosphati-dylmethanol | Anionic | A2 | 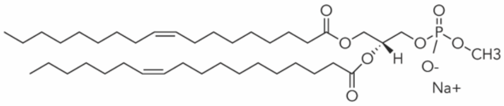 |
| 18:1 Phosphati-dylethanol | Anionic | A3 | 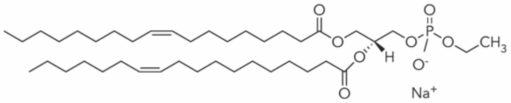 |
| 18:1 Phosphati-dylpropanol | Anionic | A4 | 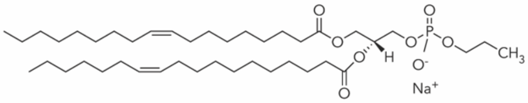 |
| DOPG | Anionic | A5 | 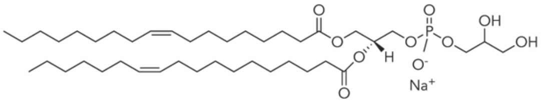 |
| DOPS | Anionic | A6 | 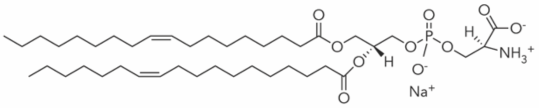 |
| 18:1 Biotinyl PE | Anionic | A7 | 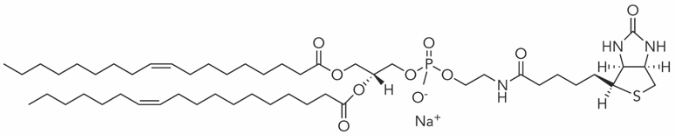 |

**Supplementary Figure 2.** Helper lipids used in the (a) cationic, (b) neutral, and (c) anionic libraries.

a

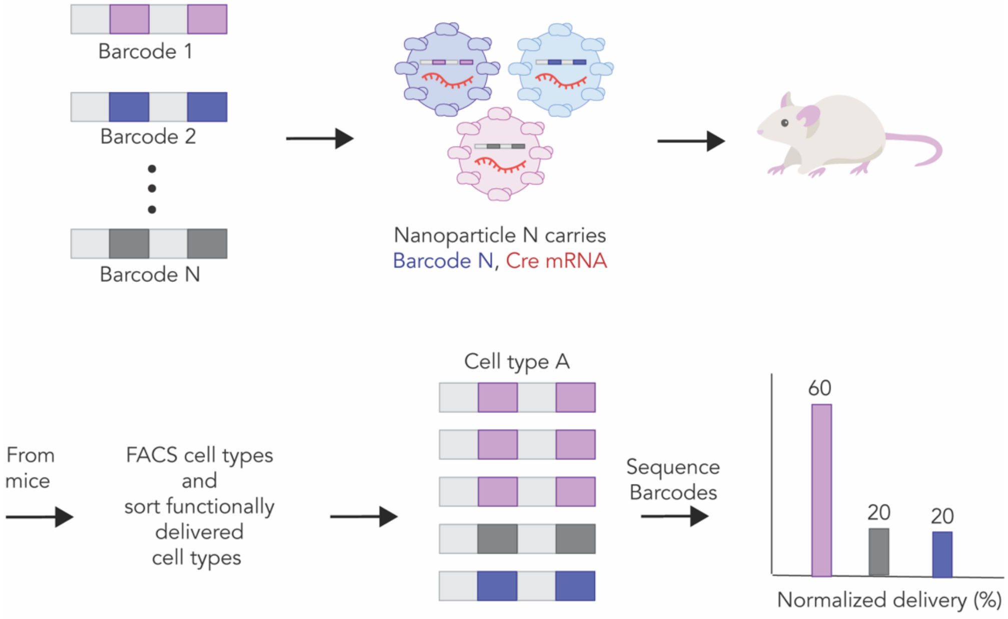


b

| Cell Types | Flow Markers |
|---|---|
| Liver Hepatocytes | CD31- CD45- |
| Liver Endothelial Cells | CD31+ CD45- |
| Liver Dendritic Cells | CD31- CD45+ CD11c+ |
| Liver T Cells | CD31- CD45+ CD3+ |
| Liver B Cells | CD31- CD45+ CD19+ |
| Liver Kupffer Cells | CD31- CD45+ CD68+ |
| Lung Endothelial Cells | CD31+ CD45- |
| Lung Dendritic Cells | CD31- CD45+ CD11c+ |
| Lung T Cells | CD31- CD45+ CD3+ |
| Lung B Cells | CD31- CD45+ CD19+ |
| Lung Monocytes | CD31- CD45+ CD11b+ |
| Spleen Dendritic Cells | CD45+ CD11c+ |
| Spleen T Cells | CD45+ CD3+ |
| Spleen B Cells | CD45+ CD19+ |
| Spleen Monocytes | CD45+ CD11b+ |
| Heart Endothelial Cells | CD31+ CD45- |
| Heart Immune Cells | CD31- CD45+ |
| Kidney Endothelial Cells | CD31+ CD45- |
| Kidney Immune Cells | CD31- CD45+ |

**Supplementary Figure 3.** (a) Example schematic of normalized delivery. (b) Cell type-specific markers for tdTomato expression measurement and sorting.

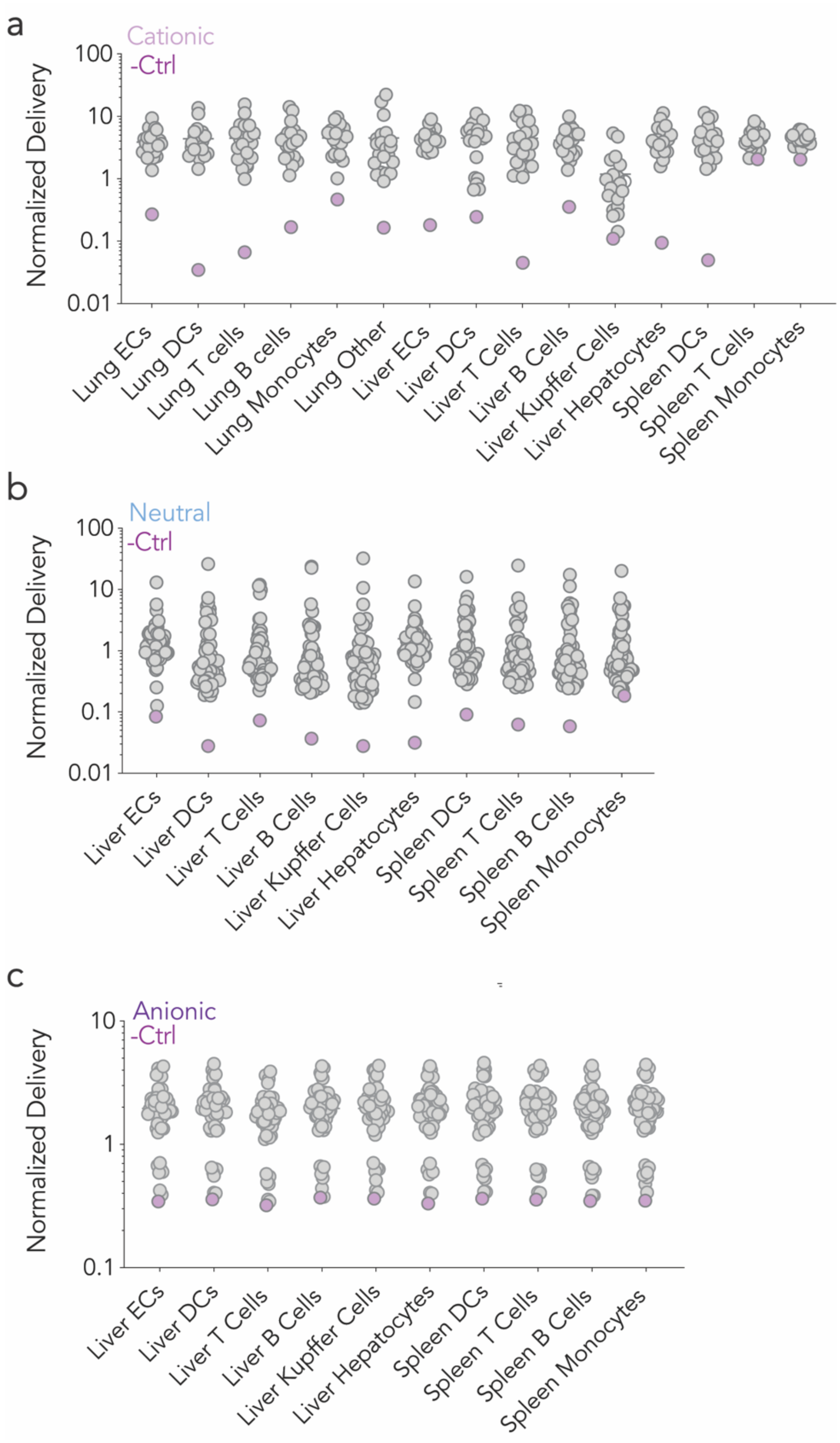
a
Cationic
-Ctrl
Normalized Delivery
100
10
1
0.1
0.01
Lung ECs
Lung DCs
Lung T cells
Lung B cells
Lung Monocytes
Lung Other
Liver ECs
Liver DCs
Liver T Cells
Liver B Cells
Liver Kupffer Cells
Liver Hepatocytes
Spleen DCs
Spleen T Cells
Spleen Monocytes
b
Neutral
-Ctrl
Normalized Delivery
100
10
1
0.1
0.01
Liver ECs
Liver DCs
Liver T Cells
Liver B Cells
Liver Kupffer Cells
Liver Hepatocytes
Spleen DCs
Spleen T Cells
Spleen B Cells
Spleen Monocytes
c
Anionic
-Ctrl
Normalized Delivery
10
1
0.1
Liver ECs
Liver DCs
Liver T Cells
Liver B Cells
Liver Kupffer Cells
Liver Hepatocytes
Spleen DCs
Spleen T Cells
Spleen B Cells
Spleen Monocytes

**Supplementary Figure 4.** Normalized delivery of all LNPs for various cell types. The negative control, unencapsulated DNA barcode, delivered less efficiently than barcodes delivered by LNPs in the (a) cationic, (b) neutral, and (c) anionic screens.

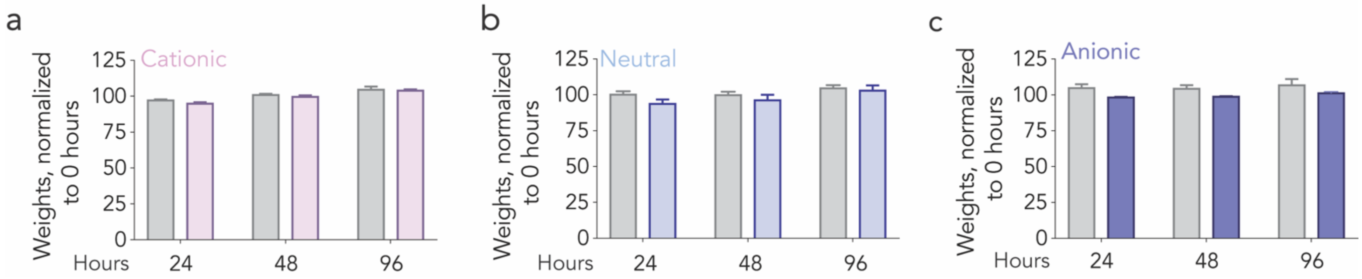


**Supplementary Figure 5.** Mouse weights measured 24, 48, and 96 hours after administration of PBS or (a) cationic, (b) neutral, and (c) anionic LNPs carrying Cre mRNA and DNA barcodes. Average +/- SEM.

a

| Barcodes | # LNP | Liver average | Spleen average | Average normalized delivery | Ionizable lipid | cholesterol | PEG | Helper lipid | Charge | Ionizable lipid molar ratio | Cholesterol Molar ratio | PEG molar ratio | Helper lipid molar ratio |
|---|---|---|---|---|---|---|---|---|---|---|---|---|---|
| AGGCGCTA | 25 | 0.861818628 | 0.553721085 | 0.707769857 | cKK-E12 | Cholesterol | C14PEG2K | DOPE | Neutral | 60 | 26 | 2 | 12 |
| CTCCTTCG | 26 | 2.68312333 | 4.300131754 | 3.491627542 | cKK-E12 | Cholesterol | C14PEG2K | DOPE | Neutral | 50 | 35 | 2.5 | 12.5 |
| GGCGCCAA | 27 | 0.825177677 | 0.739277074 | 0.782227375 | cKK-E12 | Cholesterol | C14PEG2K | DOPE | Neutral | 35 | 18 | 2.5 | 44.5 |
| TATGCCTT | 28 | 0.734607248 | 0.73938376 | 0.736995504 | cKK-E12 | Cholesterol | C14PEG2K | DOPE | Neutral | 20 | 50 | 2.5 | 27.5 |
| ATGGTAGA | 30 | 0.649198486 | 0.51607998 | 0.582639233 | cKK-E12 | Cholesterol | C14PEG2K | DSPC | Neutral | 50 | 35 | 2.5 | 12.5 |
| TGGAGACG | 31 | 0.55285347 | 0.85151389 | 0.70218368 | cKK-E12 | Cholesterol | C14PEG2K | DSPC | Neutral | 35 | 18 | 2.5 | 44.5 |
| CCGCAGAG | 32 | 16.53876183 | 19.60354139 | 18.07115161 | cKK-E12 | Cholesterol | C14PEG2K | DSPC | Neutral | 20 | 50 | 2.5 | 27.5 |
| GATCTACC | 33 | 0.721596127 | 0.404759536 | 0.563177832 | cKK-E12 | Cholesterol | C14PEG2K | 18:1 CAP PE | Neutral | 60 | 26 | 2 | 12 |
| ACGCTAGC | 34 | 4.068538713 | 5.875919068 | 4.97222889 | cKK-E12 | Cholesterol | C14PEG2K | 18:1 CAP PE | Neutral | 50 | 35 | 2.5 | 12.5 |
| TAGATCCG | 35 | 0.614616827 | 0.589923423 | 0.602270125 | cKK-E12 | Cholesterol | C14PEG2K | 18:1 CAP PE | Neutral | 35 | 18 | 2.5 | 44.5 |
| GTAATTGC | 36 | 0.685777659 | 0.548167358 | 0.616972508 | cKK-E12 | Cholesterol | C14PEG2K | 18:1 CAP PE | Neutral | 20 | 50 | 2.5 | 27.5 |
| GAGAGTTG | 37 | 0.666164327 | 0.614687633 | 0.64042598 | cKK-E12 | Cholesterol | C14PEG2K | 18:1 Lyso PC | Neutral | 60 | 26 | 2 | 12 |
| TCCAGGCG | 38 | 0.569927146 | 0.609754876 | 0.589841011 | cKK-E12 | Cholesterol | C14PEG2K | 18:1 Lyso PC | Neutral | 50 | 35 | 2.5 | 12.5 |
| ATTAGGAC | 39 | 0.681875772 | 0.643802241 | 0.662839007 | cKK-E12 | Cholesterol | C14PEG2K | 18:1 Lyso PC | Neutral | 35 | 18 | 2.5 | 44.5 |
| CTACTGAT | 41 | 0.763248889 | 0.495121701 | 0.629185295 | cKK-E12 | Cholesterol | C14PEG2K | 18:1 Dimethyl PE | Neutral | 60 | 26 | 2 | 12 |
| GCAGGACT | 42 | 0.945473037 | 1.035068032 | 0.990270535 | cKK-E12 | Cholesterol | C14PEG2K | 18:1 Dimethyl PE | Neutral | 50 | 35 | 2.5 | 12.5 |
| CGAGCAGC | 43 | 0.784297957 | 0.664964075 | 0.724631016 | cKK-E12 | Cholesterol | C14PEG2K | 18:1 Dimethyl PE | Neutral | 35 | 18 | 2.5 | 44.5 |
| GTCTCCGT | 44 | 1.137339399 | 2.29912607 | 1.718232735 | cKK-E12 | Cholesterol | C14PEG2K | 18:1 Dimethyl PE | Neutral | 15 | 55 | 2.5 | 27.5 |
| CATAATAG | 45 | 0.770335336 | 0.899045726 | 0.834690531 | cKK-E12 | Cholesterol | C14PEG2K | 18:1 Lyso PE | Neutral | 60 | 26 | 2 | 12 |
| CTCAGCAT | 46 | 0.558470732 | 0.677352235 | 0.617911483 | cKK-E12 | Cholesterol | C14PEG2K | 18:1 Lyso PE | Neutral | 50 | 35 | 2.5 | 12.5 |
| ATCAATTG | 47 | 0.529532767 | 0.423949347 | 0.476741057 | cKK-E12 | Cholesterol | C14PEG2K | 18:1 Lyso PE | Neutral | 35 | 18 | 2.5 | 44.5 |
| TTATTAAT | 48 | 1.020356455 | 0.93418106 | 0.977268758 | cKK-E12 | Cholesterol | C14PEG2K | 18:1 Lyso PE | Neutral | 15 | 55 | 2.5 | 27.5 |
| TGATCTAT | 49 | 0.956863938 | 0.637220531 | 0.797042235 | cKK-E12 | Cholesterol | C14PEG2K | 18:1 Monomethyl PE | Neutral | 60 | 26 | 2 | 12 |
| ATTGCTCT | 50 | 2.45632448 | 3.451525536 | 2.953925008 | cKK-E12 | Cholesterol | C14PEG2K | 18:1 Monomethyl PE | Neutral | 50 | 35 | 2.5 | 12.5 |
| TAAGATGA | 51 | 1.309864404 | 1.44747681 | 1.378670607 | cKK-E12 | Cholesterol | C14PEG2K | 18:1 Monomethyl PE | Neutral | 35 | 18 | 2.5 | 44.5 |
| GGTCGGTC | 55 | 0.598749306 | 0.353293591 | 0.476021448 | cKK-E12 | Cholesterol | C14PEG2K | DOPC | Neutral | 35 | 18 | 2.5 | 44.5 |
| AGGCTCAT | 56 | 1.113919103 | 2.128677691 | 1.621298397 | cKK-E12 | Cholesterol | C14PEG2K | DOPC | Neutral | 15 | 55 | 2.5 | 27.5 |
| ATGAGATG | 57 | 1.628513256 | 1.057576154 | 1.343044705 | cKK-E12 | Cholesterol | C18PEG2K | DOPE | Neutral | 60 | 26 | 2 | 12 |
| TACGCTCG | 58 | 4.377292326 | 4.468662412 | 4.422977369 | cKK-E12 | Cholesterol | C18PEG2K | DOPE | Neutral | 50 | 35 | 2.5 | 12.5 |
| AGCTCGGA | 59 | 0.88435095 | 0.543853807 | 0.714102379 | cKK-E12 | Cholesterol | C18PEG2K | DOPE | Neutral | 35 | 18 | 2.5 | 44.5 |
| AGTCCGGT | 60 | 0.852707933 | 0.720587767 | 0.78664785 | cKK-E12 | Cholesterol | C18PEG2K | DOPE | Neutral | 15 | 55 | 2.5 | 27.5 |
| AAGTCTAG | 61 | 0.674616596 | 0.770961456 | 0.722789026 | cKK-E12 | Cholesterol | C18PEG2K | DSPC | Neutral | 60 | 26 | 2 | 12 |
| AATGCTAC | 62 | 1.084793827 | 0.572019213 | 0.82840652 | cKK-E12 | Cholesterol | C18PEG2K | DSPC | Neutral | 50 | 35 | 2.5 | 12.5 |
| TTGGATCC | 63 | 1.656870446 | 1.416021919 | 1.536446183 | cKK-E12 | Cholesterol | C18PEG2K | DSPC | Neutral | 35 | 18 | 2.5 | 44.5 |
| TAGTACGT | 64 | 5.779151285 | 7.457666771 | 6.618409028 | cKK-E12 | Cholesterol | C18PEG2K | DSPC | Neutral | 15 | 55 | 2.5 | 27.5 |
| GCGAATTC | 65 | 0.743047533 | 0.429296234 | 0.586171884 | cKK-E12 | Cholesterol | C18PEG2K | 18:1 CAP PE | Neutral | 60 | 26 | 2 | 12 |
| ACGCTCCA | 66 | 2.4699494 | 4.046348488 | 3.258148944 | cKK-E12 | Cholesterol | C18PEG2K | 18:1 CAP PE | Neutral | 50 | 35 | 2.5 | 12.5 |
| TAACCGAA | 67 | 0.590231894 | 0.316801289 | 0.453516592 | cKK-E12 | Cholesterol | C18PEG2K | 18:1 CAP PE | Neutral | 35 | 18 | 2.5 | 44.5 |
| TCCTGATG | 68 | 0.801946901 | 0.736394821 | 0.769170861 | cKK-E12 | Cholesterol | C18PEG2K | 18:1 CAP PE | Neutral | 15 | 55 | 2.5 | 27.5 |
| ATTCGAGA | 69 | 0.462226071 | 0.420704907 | 0.441465489 | cKK-E12 | Cholesterol | C18PEG2K | 18:1 Lyso PC | Neutral | 60 | 26 | 2 | 12 |
| CGCGAGGC | 70 | 0.440850955 | 0.317545278 | 0.379198117 | cKK-E12 | Cholesterol | C18PEG2K | 18:1 Lyso PC | Neutral | 50 | 35 | 2.5 | 12.5 |
| ATTGGTTC | 71 | 0.353390538 | 0.257891876 | 0.305641207 | cKK-E12 | Cholesterol | C18PEG2K | 18:1 Lyso PC | Neutral | 35 | 18 | 2.5 | 44.5 |
| AATATACG | 72 | 2.208160021 | 3.469532285 | 2.838846153 | cKK-E12 | Cholesterol | C18PEG2K | 18:1 Lyso PC | Neutral | 15 | 55 | 2.5 | 27.5 |
| GATTCCGG | 73 | 1.370532068 | 1.083444048 | 1.226988058 | cKK-E12 | Cholesterol | C18PEG2K | 18:1 Dimethyl PE | Neutral | 60 | 26 | 2 | 12 |
| CGGTCAAT | 74 | 0.984142614 | 1.567468847 | 1.27580573 | cKK-E12 | Cholesterol | C18PEG2K | 18:1 Dimethyl PE | Neutral | 50 | 35 | 2.5 | 12.5 |
| TATATCTA | 75 | 11.46422587 | 0.783457322 | 6.123841597 | cKK-E12 | Cholesterol | C18PEG2K | 18:1 Dimethyl PE | Neutral | 35 | 18 | 2.5 | 44.5 |
| ATCGTCTA | 76 | 0.207095482 | 0.34433757 | 0.275716526 | cKK-E12 | Cholesterol | C18PEG2K | 18:1 Dimethyl PE | Neutral | 15 | 55 | 2.5 | 27.5 |
| CTACCATT | 77 | 0.47598737 | 0.382180081 | 0.429083725 | cKK-E12 | Cholesterol | C18PEG2K | 18:1 Lyso PE | Neutral | 60 | 26 | 2 | 12 |
| GTCGAAGT | 78 | 0.410570173 | 0.429370102 | 0.419970137 | cKK-E12 | Cholesterol | C18PEG2K | 18:1 Lyso PE | Neutral | 50 | 35 | 2.5 | 12.5 |
| GATGGCCT | 79 | 0.429523374 | 0.426689046 | 0.42810621 | cKK-E12 | Cholesterol | C18PEG2K | 18:1 Lyso PE | Neutral | 35 | 18 | 2.5 | 44.5 |
| CGATGCTT | 80 | 2.033517386 | 2.938566917 | 2.486042152 | cKK-E12 | Cholesterol | C18PEG2K | 18:1 Lyso PE | Neutral | 15 | 55 | 2.5 | 27.5 |
| ATAATATA | 81 | 0.792989213 | 1.889577885 | 1.341283549 | cKK-E12 | Cholesterol | C18PEG2K | 18:1 Monomethyl PE | Neutral | 60 | 26 | 2 | 12 |
| CGCCTATT | 82 | 3.141775937 | 5.041427408 | 4.091601673 | cKK-E12 | Cholesterol | C18PEG2K | 18:1 Monomethyl PE | Neutral | 50 | 35 | 2.5 | 12.5 |
| AAGAGGAT | 83 | 1.355188536 | 0.649279953 | 1.002234244 | cKK-E12 | Cholesterol | C18PEG2K | 18:1 Monomethyl PE | Neutral | 35 | 18 | 2.5 | 44.5 |
| GGACGTCC | 84 | 0.894417467 | 0.491410264 | 0.692913866 | cKK-E12 | Cholesterol | C18PEG2K | 18:1 Monomethyl PE | Neutral | 15 | 55 | 2.5 | 27.5 |
| GTTAGTCA | 85 | 1.271284929 | 0.334964168 | 0.803124549 | cKK-E12 | Cholesterol | C18PEG2K | DOPC | Neutral | 60 | 26 | 2 | 12 |
| ACTATCTC | 86 | 0.781952094 | 0.644871119 | 0.713411606 | cKK-E12 | Cholesterol | C18PEG2K | DOPC | Neutral | 50 | 35 | 2.5 | 12.5 |
| TTATAGCA | 87 | 0.440182205 | 0.27199457 | 0.356088388 | cKK-E12 | Cholesterol | C18PEG2K | DOPC | Neutral | 35 | 18 | 2.5 | 44.5 |
| TGACCAGG | Naked Barcode | 0.046855224 | 0.098606238 | 0.072730731 | | | | | | | | | |

b

| Barcode | LNP # | Liver average | Spleen average | average normalized delivery | Ionizable lipid | Cholesterol | PEG | Helper lipid | Charge | Ionizable lipid molar ratio | Cholesterol molar ratio | PEG molar ratio | Helper lipid molar ratio |
|---|---|---|---|---|---|---|---|---|---|---|---|---|---|
| GACGCAAT | 1 | 4.021957523 | 4.034426393 | 4.028191958 | cKK-E12 | Cholesterol | C14PEG2K | 18:1 Phosphatidylethanol | Anionic | 60 | 26 | 2 | 12 |
| GATTCAAC | 2 | 2.378888101 | 2.364877116 | 2.371882609 | cKK-E12 | Cholesterol | C14PEG2K | 18:1 Phosphatidylethanol | Anionic | 50 | 35 | 2.5 | 12.5 |
| TCCTAAGA | 3 | 2.592904106 | 2.590034791 | 2.591469449 | cKK-E12 | Cholesterol | C14PEG2K | 18:1 Phosphatidylethanol | Anionic | 35 | 18 | 2.5 | 44.5 |
| CATCATTA | 4 | 2.312457781 | 2.312064813 | 2.312261297 | cKK-E12 | Cholesterol | C14PEG2K | 18:1 Phosphatidylethanol | Anionic | 20 | 50 | 2.5 | 27.5 |
| CAATCCGT | 5 | 2.179047208 | 2.190960732 | 2.18500397 | cKK-E12 | Cholesterol | C14PEG2K | 18:1 Phosphatidylpropanol | Anionic | 60 | 26 | 2 | 12 |
| CTCAACTA | 6 | 1.792971437 | 1.796966775 | 1.794969106 | cKK-E12 | Cholesterol | C14PEG2K | 18:1 Phosphatidylpropanol | Anionic | 50 | 35 | 2.5 | 12.5 |
| AGTTACCG | 7 | 2.024783075 | 2.021071307 | 2.022927191 | cKK-E12 | Cholesterol | C14PEG2K | 18:1 Phosphatidylpropanol | Anionic | 35 | 18 | 2.5 | 44.5 |
| CGCTCCGG | 8 | 1.613075135 | 1.610757019 | 1.611916077 | cKK-E12 | Cholesterol | C14PEG2K | 18:1 Phosphatidylpropanol | Anionic | 20 | 50 | 2.5 | 27.5 |
| GCGAGTAT | 9 | 1.882683403 | 1.910537481 | 1.896610442 | cKK-E12 | Cholesterol | C14PEG2K | 18:1 Phosphatidylmethanol | Anionic | 60 | 26 | 2 | 12 |
| TACCTGCT | 10 | 1.932287369 | 1.927959638 | 1.930123504 | cKK-E12 | Cholesterol | C14PEG2K | 18:1 Phosphatidylmethanol | Anionic | 50 | 35 | 2.5 | 12.5 |
| CAAGAAGG | 11 | 2.726414256 | 2.725420218 | 2.725917237 | cKK-E12 | Cholesterol | C14PEG2K | 18:1 Phosphatidylmethanol | Anionic | 35 | 18 | 2.5 | 44.5 |
| TTGCAACT | 12 | 0.406247919 | 0.413464049 | 0.409855984 | cKK-E12 | Cholesterol | C14PEG2K | 18:1 Phosphatidylmethanol | Anionic | 20 | 50 | 2.5 | 27.5 |
| GGCGCTTG | 13 | 1.383450969 | 1.381216911 | 1.38233394 | cKK-E12 | Cholesterol | C14PEG2K | 18:1 (Δ9-Cis) PG | Anionic | 60 | 26 | 2 | 12 |
| CTTACGTC | 14 | 2.406492122 | 2.397056946 | 2.401774534 | cKK-E12 | Cholesterol | C14PEG2K | 18:1 (Δ9-Cis) PG | Anionic | 50 | 35 | 2.5 | 12.5 |
| GAGTATAC | 15 | 1.891865534 | 1.895610456 | 1.893737995 | cKK-E12 | Cholesterol | C14PEG2K | 18:1 (Δ9-Cis) PG | Anionic | 35 | 18 | 2.5 | 44.5 |
| ACTCAAGT | 16 | 0.654826991 | 0.656881301 | 0.655854146 | cKK-E12 | Cholesterol | C14PEG2K | 18:1 (Δ9-Cis) PG | Anionic | 20 | 50 | 2.5 | 27.5 |
| ACCTAATC | 17 | 4.028778928 | 4.009837145 | 4.019308036 | cKK-E12 | Cholesterol | C14PEG2K | 18:1 PA | Anionic | 60 | 26 | 2 | 12 |
| GCTAATCG | 18 | 3.538539914 | 3.538066598 | 3.538303256 | cKK-E12 | Cholesterol | C14PEG2K | 18:1 PA | Anionic | 50 | 35 | 2.5 | 12.5 |
| TAGAATTA | 19 | 3.662188138 | 3.664231961 | 3.663210049 | cKK-E12 | Cholesterol | C14PEG2K | 18:1 PA | Anionic | 35 | 18 | 2.5 | 44.5 |
| GTCCGTTA | 21 | 1.412308546 | 1.408956732 | 1.410632639 | cKK-E12 | Cholesterol | C14PEG2K | 18:1 PS (DOPS) | Anionic | 60 | 26 | 2 | 12 |
| TGAGTTCG | 22 | 2.321968887 | 2.316624711 | 2.319296799 | cKK-E12 | Cholesterol | C14PEG2K | 18:1 PS (DOPS) | Anionic | 50 | 35 | 2.5 | 12.5 |
| GGCAGTAG | 23 | 1.661131669 | 1.655391318 | 1.658261493 | cKK-E12 | Cholesterol | C14PEG2K | 18:1 PS (DOPS) | Anionic | 35 | 18 | 2.5 | 44.5 |
| CCGTTCGG | 24 | 2.433786524 | 2.426291791 | 2.430039158 | cKK-E12 | Cholesterol | C14PEG2K | 18:1 PS (DOPS) | Anionic | 20 | 50 | 2.5 | 27.5 |
| AGGCGCTA | 25 | 2.332935032 | 2.329069744 | 2.331002388 | cKK-E12 | Cholesterol | C14PEG2K | 18:1 Biotinyl PE | Anionic | 60 | 26 | 2 | 12 |
| CTCCTTCG | 26 | 2.635473127 | 2.62850754 | 2.631990334 | cKK-E12 | Cholesterol | C14PEG2K | 18:1 Biotinyl PE | Anionic | 50 | 35 | 2.5 | 12.5 |
| GGCGCCAA | 27 | 2.761871407 | 2.753355469 | 2.757613438 | cKK-E12 | Cholesterol | C14PEG2K | 18:1 Biotinyl PE | Anionic | 35 | 18 | 2.5 | 44.5 |
| TATGCCTT | 28 | 1.888828973 | 1.900086833 | 1.894457903 | cKK-E12 | Cholesterol | C14PEG2K | 18:1 Biotinyl PE | Anionic | 20 | 50 | 2.5 | 27.5 |
| GATCTACC | 29 | 0.618966493 | 0.621760326 | 0.62036341 | cKK-E12 | Cholesterol | C18PEG2K | 18:1 Phosphatidylethanol | Anionic | 60 | 26 | 2 | 12 |
| ACGCTAGC | 30 | 1.692227523 | 1.693133964 | 1.692680744 | cKK-E12 | Cholesterol | C18PEG2K | 18:1 Phosphatidylethanol | Anionic | 50 | 35 | 2.5 | 12.5 |
| TAGATCCG | 31 | 1.242107808 | 1.247332471 | 1.24472014 | cKK-E12 | Cholesterol | C18PEG2K | 18:1 Phosphatidylethanol | Anionic | 35 | 18 | 2.5 | 44.5 |
| GTAATTGC | 32 | 0.400936326 | 0.401517061 | 0.401226693 | cKK-E12 | Cholesterol | C18PEG2K | 18:1 Phosphatidylethanol | Anionic | 20 | 50 | 2.5 | 27.5 |
| GAGAGTTG | 33 | 1.623050794 | 1.622838247 | 1.62294452 | cKK-E12 | Cholesterol | C18PEG2K | 18:1 Phosphatidylpropanol | Anionic | 60 | 26 | 2 | 12 |
| CGGTATCT | 36 | 1.412293735 | 1.420897262 | 1.416595498 | cKK-E12 | Cholesterol | C18PEG2K | 18:1 Phosphatidylpropanol | Anionic | 20 | 50 | 2.5 | 27.5 |
| GCAGGACT | 38 | 2.460927482 | 2.453570895 | 2.457249188 | cKK-E12 | Cholesterol | C18PEG2K | 18:1 Phosphatidylmethanol | Anionic | 50 | 35 | 2.5 | 12.5 |
| CGAGCAGC | 39 | 1.975416712 | 1.969808923 | 1.972612817 | cKK-E12 | Cholesterol | C18PEG2K | 18:1 Phosphatidylmethanol | Anionic | 35 | 18 | 2.5 | 44.5 |
| GTCTCCGT | 40 | 1.692249013 | 1.694979096 | 1.693614054 | cKK-E12 | Cholesterol | C18PEG2K | 18:1 Phosphatidylmethanol | Anionic | 20 | 50 | 2.5 | 27.5 |
| CATAATAG | 41 | 2.113006136 | 2.108318603 | 2.110662369 | cKK-E12 | Cholesterol | C18PEG2K | 18:1 (Δ9-Cis) PG | Anionic | 60 | 26 | 2 | 12 |
| CTCAGCAT | 42 | 0.53775489 | 0.537953146 | 0.537854018 | cKK-E12 | Cholesterol | C18PEG2K | 18:1 (Δ9-Cis) PG | Anionic | 50 | 35 | 2.5 | 12.5 |
| ATCAATTG | 43 | 1.845686044 | 1.843759497 | 1.844722771 | cKK-E12 | Cholesterol | C18PEG2K | 18:1 (Δ9-Cis) PG | Anionic | 35 | 18 | 2.5 | 44.5 |
| TTATTAAT | 44 | 1.401164973 | 1.414561708 | 1.407863341 | cKK-E12 | Cholesterol | C18PEG2K | 18:1 (Δ9-Cis) PG | Anionic | 20 | 50 | 2.5 | 27.5 |
| TGATCTAT | 45 | 1.963682303 | 1.954207477 | 1.95894489 | cKK-E12 | Cholesterol | C18PEG2K | 18:1 PA | Anionic | 60 | 26 | 2 | 12 |
| ATTGCTCT | 46 | 2.129405956 | 2.125857035 | 2.127631495 | cKK-E12 | Cholesterol | C18PEG2K | 18:1 PA | Anionic | 50 | 35 | 2.5 | 12.5 |
| TAAGATGA | 47 | 0.562905513 | 0.563076041 | 0.562990777 | cKK-E12 | Cholesterol | C18PEG2K | 18:1 PA | Anionic | 35 | 18 | 2.5 | 44.5 |
| TCTAGAGT | 49 | 2.321898341 | 2.32804315 | 2.324970745 | cKK-E12 | Cholesterol | C18PEG2K | 18:1 PS (DOPS) | Anionic | 60 | 26 | 2 | 12 |
| TGCGTATA | 50 | 1.776926588 | 1.779371818 | 1.778149203 | cKK-E12 | Cholesterol | C18PEG2K | 18:1 PS (DOPS) | Anionic | 50 | 35 | 2.5 | 12.5 |
| AGGCTCAT | 52 | 4.439148827 | 4.43943094 | 4.439289883 | cKK-E12 | Cholesterol | C18PEG2K | 18:1 PS (DOPS) | Anionic | 20 | 50 | 2.5 | 27.5 |
| ATGAGATG | 53 | 2.068792191 | 2.072694909 | 2.07074355 | cKK-E12 | Cholesterol | C18PEG2K | 18:1 Biotinyl PE | Anionic | 60 | 26 | 2 | 12 |
| TACGCTCG | 54 | 0.6219439 | 0.618805294 | 0.620374597 | cKK-E12 | Cholesterol | C18PEG2K | 18:1 Biotinyl PE | Anionic | 50 | 35 | 2.5 | 12.5 |
| AGCTCGGA | 55 | 2.512635853 | 2.518641331 | 2.515638592 | cKK-E12 | Cholesterol | C18PEG2K | 18:1 Biotinyl PE | Anionic | 35 | 18 | 2.5 | 44.5 |
| AGTCCGGT | 56 | 1.335397612 | 1.33884476 | 1.337121186 | cKK-E12 | Cholesterol | C18PEG2K | 18:1 Biotinyl PE | Anionic | 20 | 50 | 2.5 | 27.5 |
| TGACCAGG | Naked Barcode | 0.355311137 | 0.354670459 | 0.354990798 | | | | | | | | | |

c

| Barcodes | # LNP | Lung average | Liver average | spleen average | average normalized delivery | onizable lipid | Cholesterol | PEG | Helper lipid | Charge | Ionizable lipid molar ratio | Cholesterol Molar ratio | PEG molar ratio | Helper lipid molar ratio |
|---|---|---|---|---|---|---|---|---|---|---|---|---|---|---|
| AGCATGCG | 2 | 12.56256328 | 8.524294312 | 3.402668606 | 8.163175401 | cKK-E12 | Cholesterol | C14PEG2K | DOTAP | Cationic | 50 | 35 | 2.5 | 12.5 |
| TTGCGTTG | 3 | 2.377185354 | 1.911603866 | 4.620508024 | 2.969765748 | cKK-E12 | Cholesterol | C14PEG2K | DOTAP | Cationic | 35 | 18 | 2.5 | 44.5 |
| ACGTCGAA | 4 | 2.002639125 | 2.081597779 | 8.175763564 | 4.086666823 | cKK-E12 | Cholesterol | C14PEG2K | DOTAP | Cationic | 20 | 50 | 2.5 | 27.5 |
| CTACGAGG | 5 | 3.461523684 | 2.550574912 | 1.425292919 | 2.479130505 | cKK-E12 | Cholesterol | C14PEG2K | DOTMA | Cationic | 60 | 26 | 2 | 12 |
| ATAGAATC | 6 | 2.388169424 | 2.140902725 | 10.74356823 | 5.090880125 | cKK-E12 | Cholesterol | C14PEG2K | DOTMA | Cationic | 50 | 35 | 2.5 | 12.5 |
| GTCGCCTC | 7 | 2.072582804 | 3.047458645 | 1.858859395 | 2.326300281 | cKK-E12 | Cholesterol | C14PEG2K | DOTMA | Cationic | 35 | 18 | 2.5 | 44.5 |
| GTCAATCT | 8 | 1.496114241 | 1.40017483 | 4.971669107 | 2.622652726 | cKK-E12 | Cholesterol | C14PEG2K | DOTMA | Cationic | 20 | 50 | 2.5 | 27.5 |
| GACGCAAT | 9 | 4.559002703 | 7.725292314 | 3.311535108 | 5.198610041 | cKK-E12 | Cholesterol | C14PEG2K | DDAB | Cationic | 60 | 26 | 2 | 12 |
| GATTCAAC | 10 | 5.65587824 | 4.931457318 | 3.462286173 | 4.683207244 | cKK-E12 | Cholesterol | C14PEG2K | DDAB | Cationic | 50 | 35 | 2.5 | 12.5 |
| TCCTAAGA | 11 | 10.70795163 | 6.787428278 | 3.698774055 | 7.064717988 | cKK-E12 | Cholesterol | C14PEG2K | DDAB | Cationic | 35 | 18 | 2.5 | 44.5 |
| CATCATTA | 12 | 2.448116757 | 2.785822786 | 2.171807573 | 2.468582372 | cKK-E12 | Cholesterol | C14PEG2K | DDAB | Cationic | 20 | 50 | 2.5 | 27.5 |
| TACCTGCT | 13 | 4.239008784 | 4.491167191 | 1.415055526 | 3.381743834 | cKK-E12 | Cholesterol | C18PEG2K | DOTAP | Cationic | 50 | 35 | 2.5 | 12.5 |
| CAAGAAGG | 15 | 5.687825976 | 4.656207347 | 3.139577841 | 4.494537055 | cKK-E12 | Cholesterol | C18PEG2K | DOTAP | Cationic | 35 | 18 | 2.5 | 44.5 |
| TTGCAACT | 16 | 2.328265292 | 1.905498327 | 2.021396178 | 2.085053266 | cKK-E12 | Cholesterol | C18PEG2K | DOTAP | Cationic | 20 | 50 | 2.5 | 27.5 |
| GGCGCTTG | 17 | 4.739717288 | 3.938939394 | 4.660291473 | 4.446316052 | cKK-E12 | Cholesterol | C18PEG2K | DOTMA | Cationic | 60 | 26 | 2 | 12 |
| CTTACGTC | 18 | 3.334529063 | 2.722239283 | 7.944338444 | 4.667035596 | cKK-E12 | Cholesterol | C18PEG2K | DOTMA | Cationic | 50 | 35 | 2.5 | 12.5 |
| GAGTATAC | 19 | 4.255948685 | 3.024652113 | 2.68576884 | 3.322123213 | cKK-E12 | Cholesterol | C18PEG2K | DOTMA | Cationic | 35 | 18 | 2.5 | 44.5 |
| ACTCAAGT | 20 | 5.750342045 | 3.341275375 | 3.008190304 | 4.033269242 | cKK-E12 | Cholesterol | C18PEG2K | DOTMA | Cationic | 20 | 50 | 2.5 | 27.5 |
| ACCTAATC | 21 | 1.409544503 | 5.561151432 | 9.671625209 | 5.547440381 | cKK-E12 | Cholesterol | C18PEG2K | DDAB | Cationic | 60 | 26 | 2 | 12 |
| GCTAATCG | 22 | 5.863723904 | 5.079908054 | 4.604695188 | 5.182775715 | cKK-E12 | Cholesterol | C18PEG2K | DDAB | Cationic | 50 | 35 | 2.5 | 12.5 |
| TAGAATTA | 23 | 3.145606084 | 3.53427274 | 3.2043026 | 3.294727141 | cKK-E12 | Cholesterol | C18PEG2K | DDAB | Cationic | 35 | 18 | 2.5 | 44.5 |
| TCTAACTG | 24 | 6.763484213 | 7.222256178 | 3.218984213 | 5.734908201 | cKK-E12 | Cholesterol | C18PEG2K | DDAB | Cationic | 20 | 50 | 2.5 | 27.5 |
| TTGCCGGT | Naked Barcode | 0.11430871 | 0.163102562 | 0.033280231 | 0.103563834 | | | | | | | | | |

d

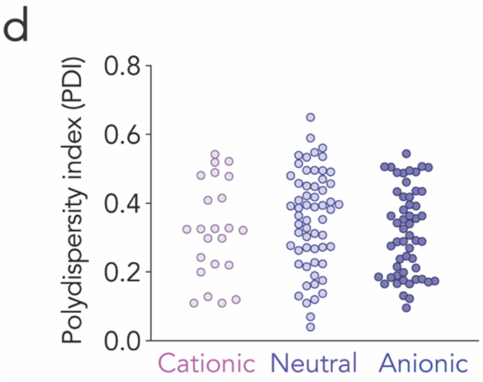


**Supplementary Figure 6.** Normalized delivery of LNPs in (a) neutral screen, (b) anionic screen, and (c) cationic screen. The lead LNPs (Neu-LNP, An-LNP, Cat-LNP in order) in each screen are shown in the red boxes. (d) Polydispersity index of all individual pooled LNPs.

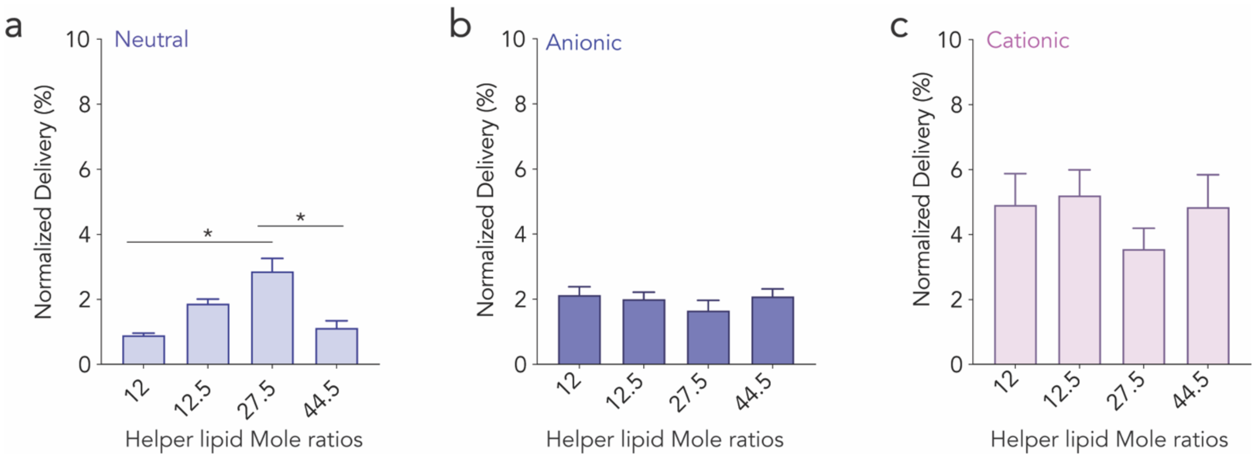


**Supplementary Figure 7.** (a) Normalized delivery of neutral LNPs was highest at 27.5% helper lipid mole ratio. (b, c) No relationship was found between helper lipid mole ratio and normalized delivery of anionic and cationic LNPs. **P* < 0.04, one-way ANOVA, average +/- SEM.

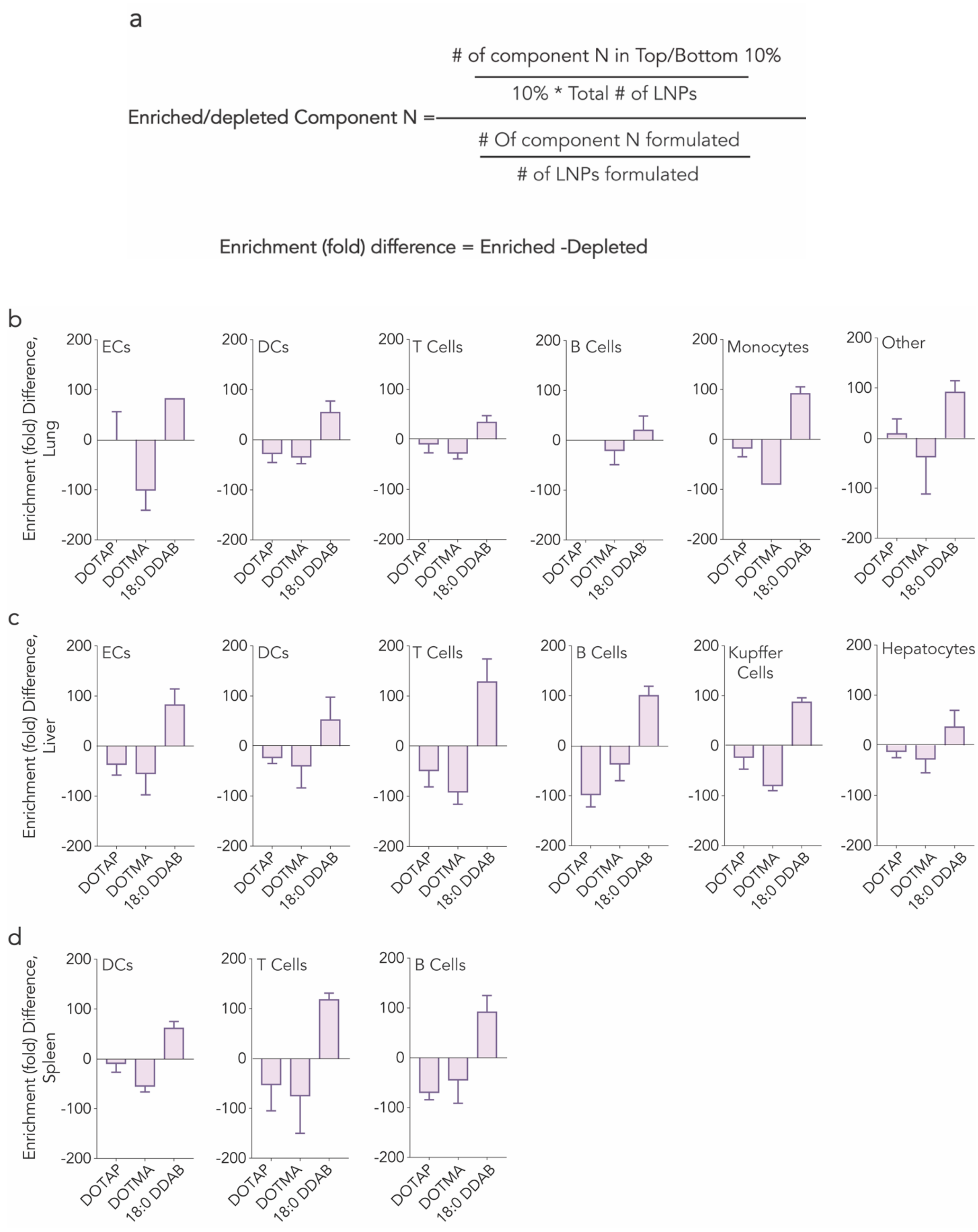


**Supplementary Figure 8**. (a) Enrichment (fold) difference is calculated through the formula shown. Fold enrichment was calculated for cationic LNPs delivered to (b) lung cells, (c) liver cells, and (d) spleen cells. Average +/- SEM.

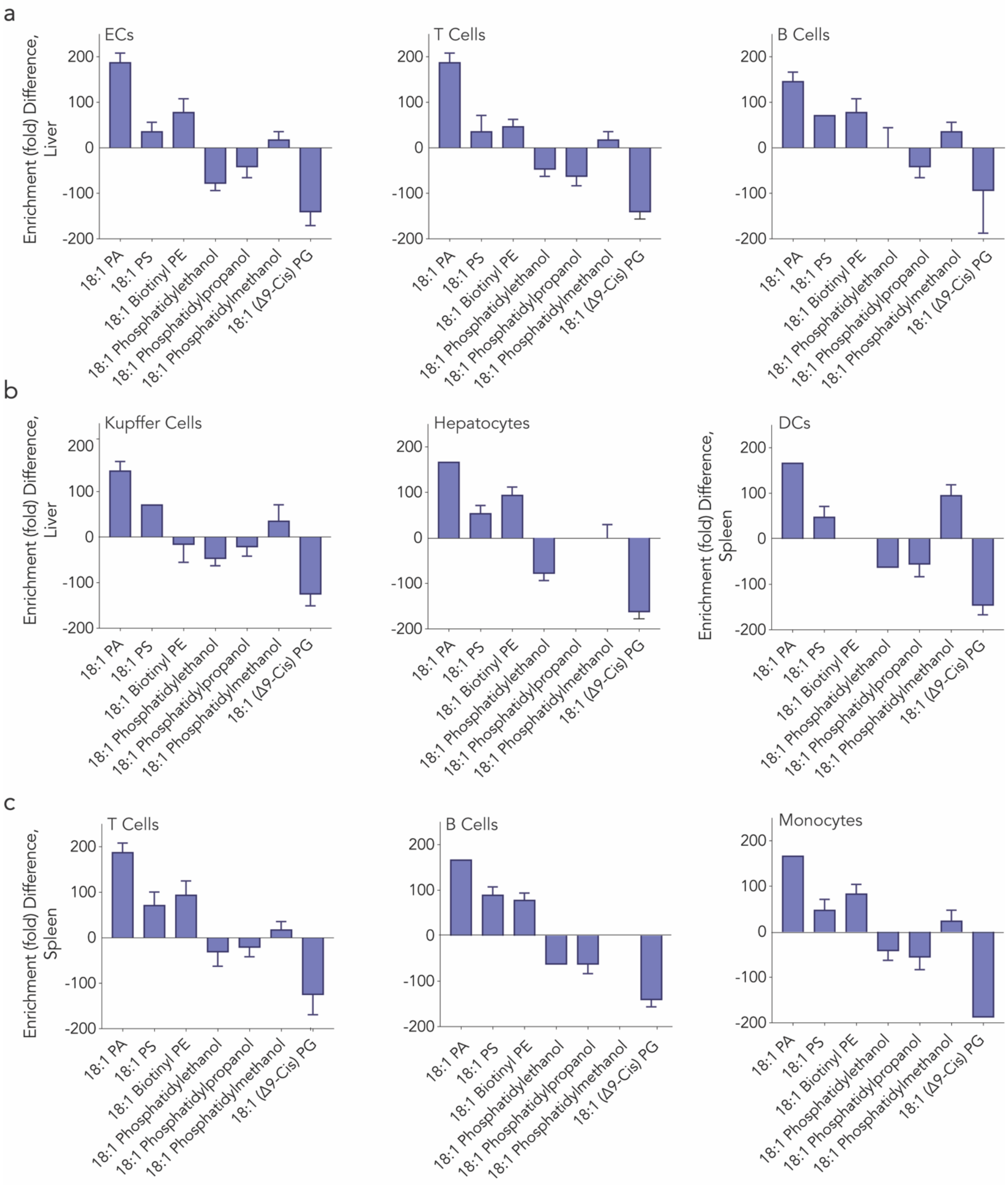


**Supplementary Figure 9**. (a, b) Fold enrichment was calculated for anionic LNPs delivered to liver cells and (c) spleen cells. Average +/- SEM.

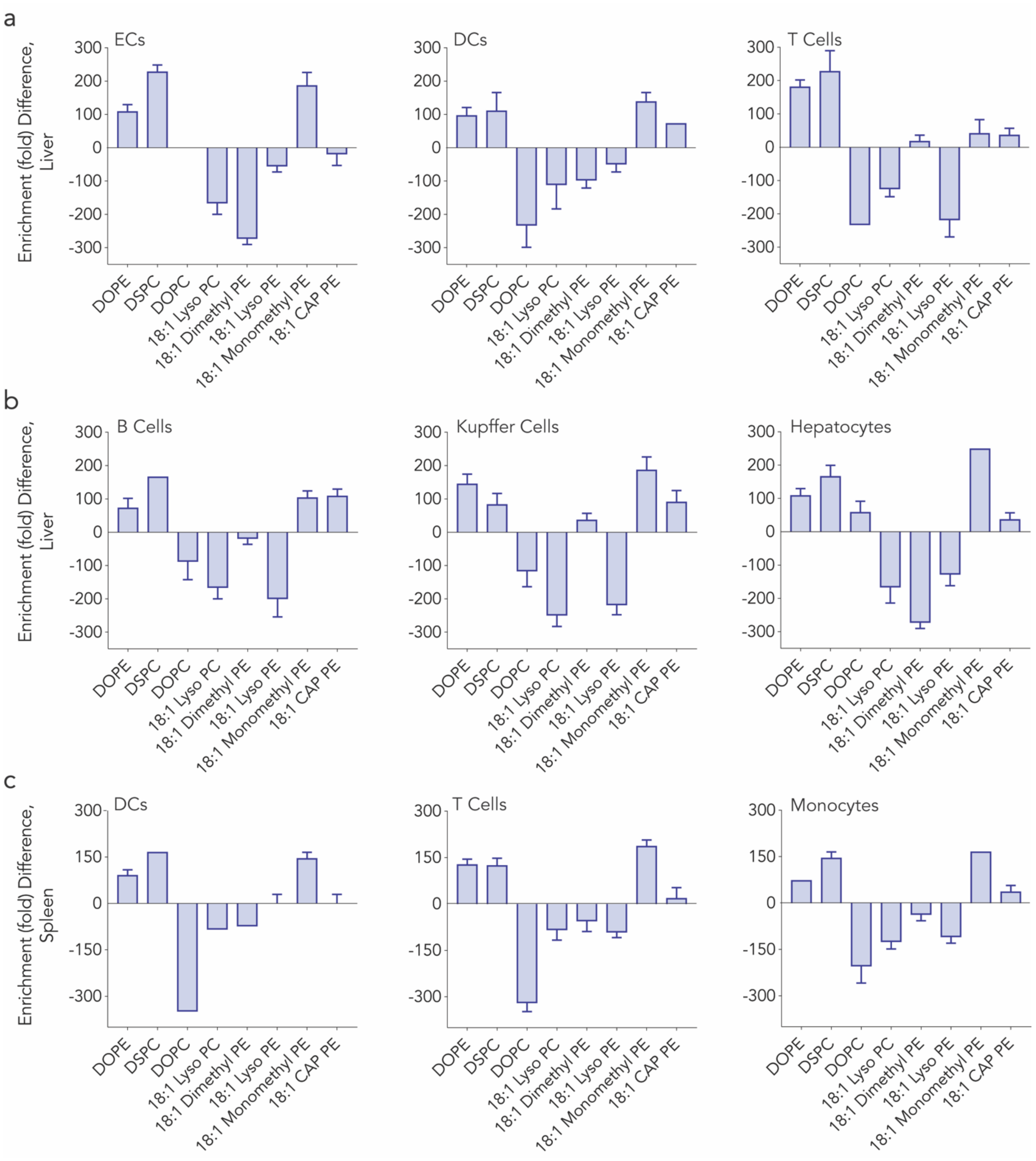


**Supplementary Figure 10**. (a, b) Fold enrichment was calculated for neutral LNPs delivered to liver cells and (c) spleen cells. Average +/- SEM.

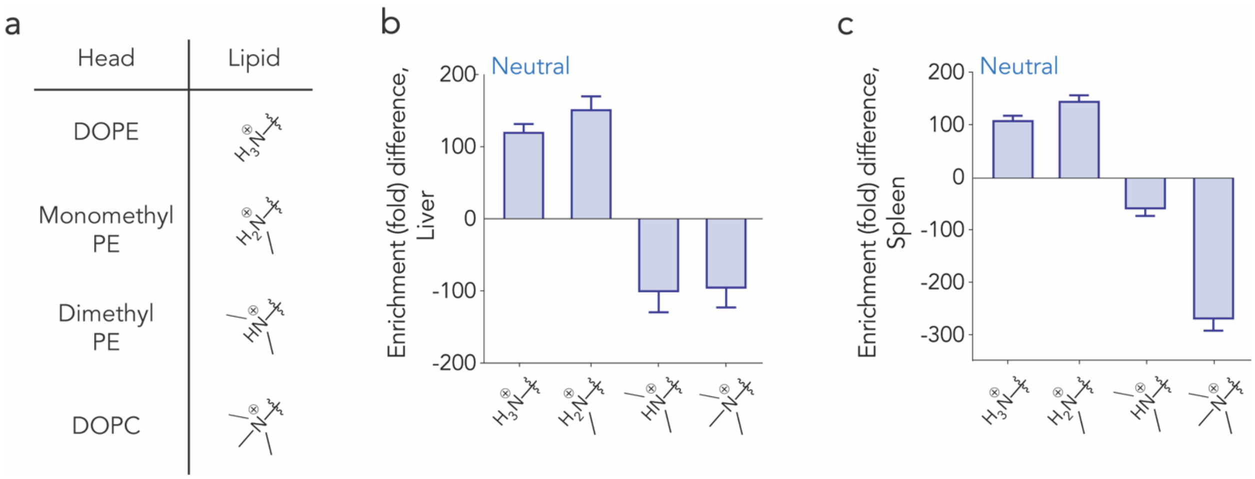


**Supplementary Figure 11.** (a) Fold enrichment in the neutral library was calculated for four different headgroups in the (b) liver and (c) spleen. Average +/- SEM.

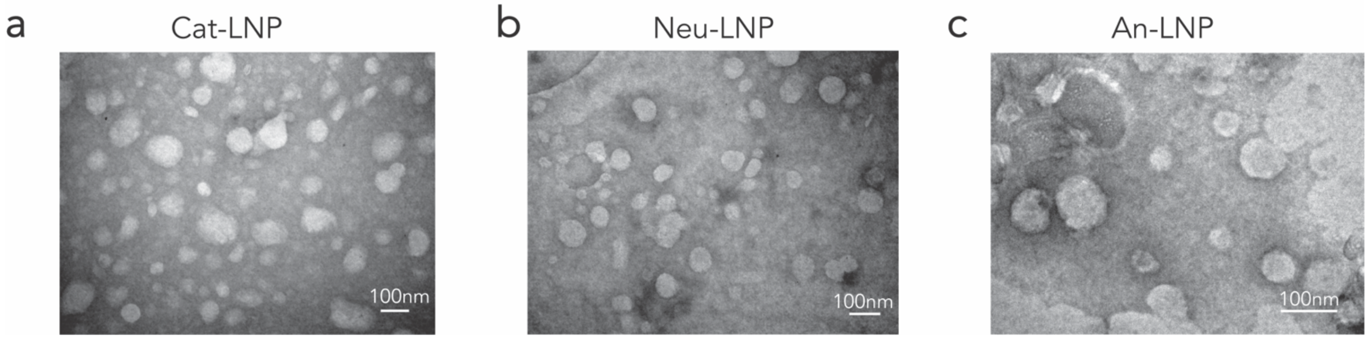


**Supplementary Figure 12.** TEM images of (a) Cat-LNP, (b) Neu-LNP, and (c) An-LNP.

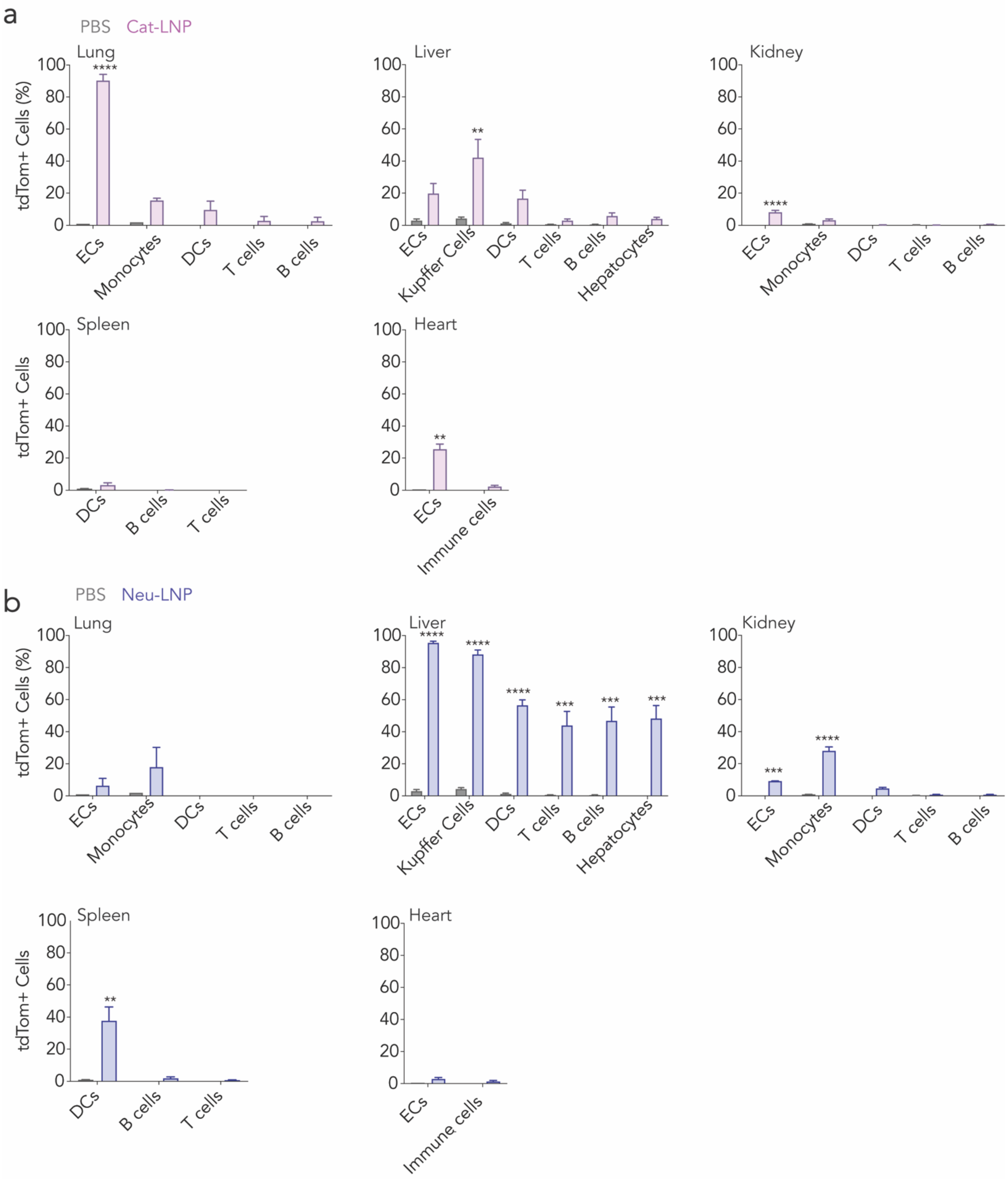
a
PBS Cat-LNP
Lung
Liver
Kidney
Spleen
Heart
tdTom+ Cells (%)
tdTom+ Cells
ECs
Monocytes
DCs
T cells
B cells
Kupffer Cells
Hepatocytes
Immune cells
b
PBS Neu-LNP

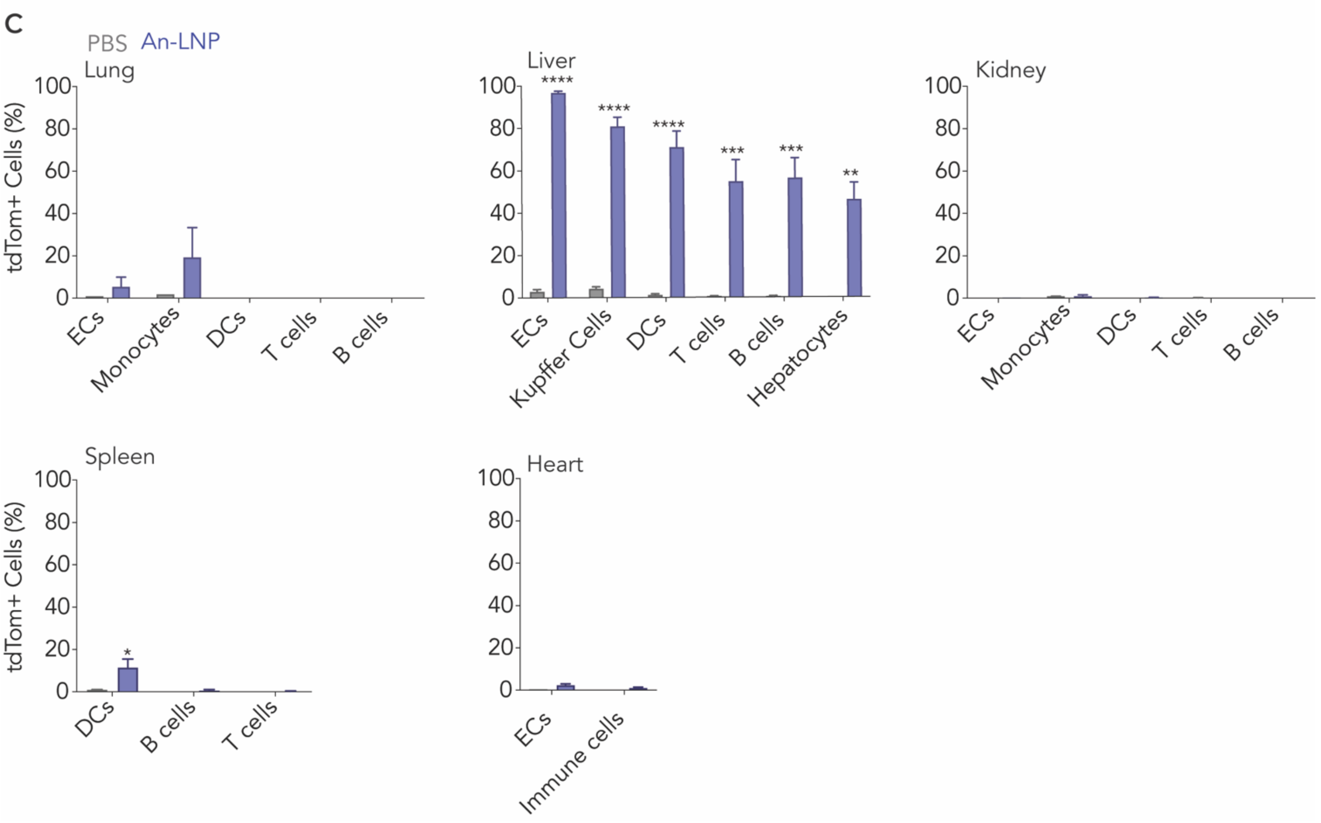


**Supplementary Figure 13.** (a) Cat-LNP enhanced functional non-liver:liver Cre mRNA delivery, with the highest delivery found in lung ECs. (b) Neu-LNP functionally delivered Cre-mRNA to liver cell types, with the most significant delivery to liver ECs and Kupffer cells. (c) An-LNP functionally delivered Cre-mRNA to liver cell types. ****$P < 0.0001$, ***$P < 0.0008$, **$P < 0.006$, *$P = 0.035$, two-way ANOVA, average +/- SEM.

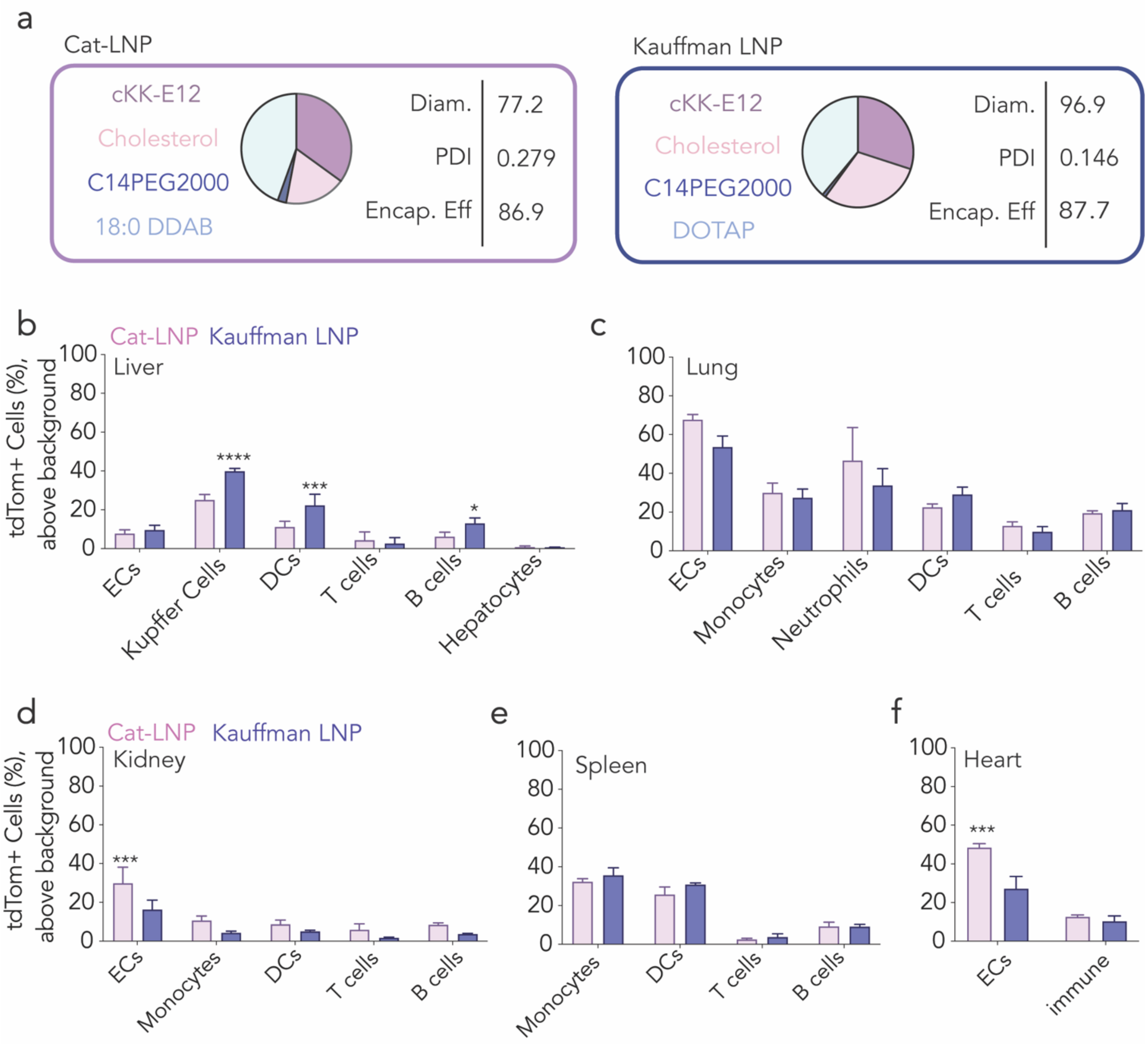


**Supplementary Figure 14.** (a) Cat-LNP and Kauffman LNP carrying Cre mRNA formed small, monodisperse particles. Diameters are in nm, and encapsulation efficiencies are in percentage. (b–f) Cat-LNP increased the ratio of non-liver to liver delivery as compared to Kauffman LNP. ****$P$ < 0.0001, ***$P$ < 0.0007, *$P$ < 0.05, two-way ANOVA, average +/- SD.

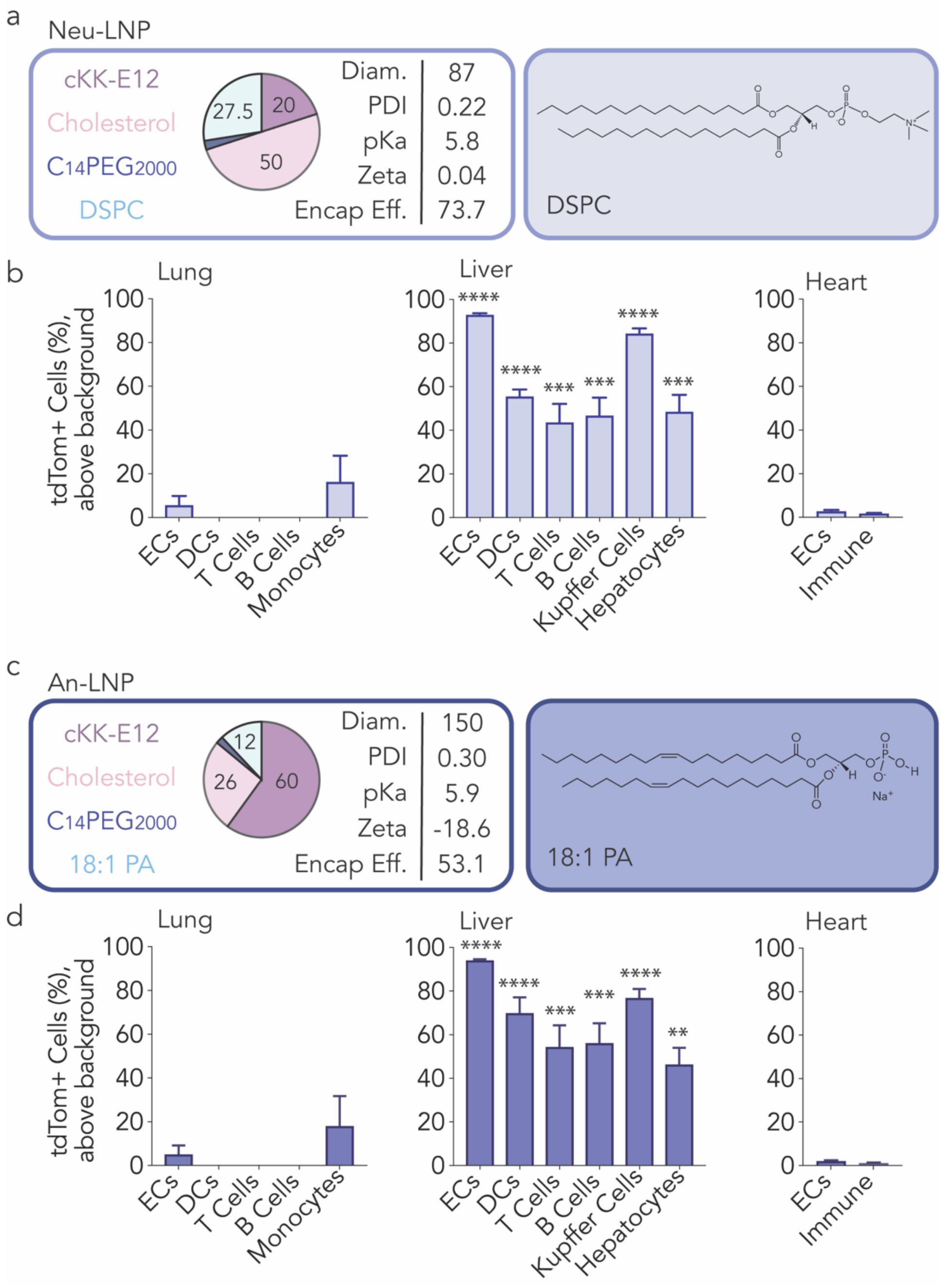


**Supplementary Figure 15.** Top LNPs from the neutral and anionic screens potently deliver mRNA to liver. (a) A lipid nanoparticle was identified from the neutral screen and named Neu-LNP. Using a neutral helper lipid, DSPC, a small and stable nanoparticle was formed. (b) The Neu-LNP was formulated with Cre mRNA and administered to Ai14 mice intravenously. Four days later, tdTomato signal was quantified. We found that the Neu-LNP delivered Cre mRNA significantly to all liver cell types. (c) An LNP was identified from the anionic screen and named An-LNP. Using an anionic helper lipid, 18:1 PA, a small and stable nanoparticle was formed. (d) The An-LNP was formulated with Cre mRNA and administered to Ai14 mice intravenously. Four days later, tdTomato signal was quantified. We found that the An-LNP delivered Cre mRNA significantly to all liver cell types. ****$P < 0.0001$, ***$P < 0.0008$, **$P = 0.0024$, two-way ANOVA, average +/- SEM. Diam.: Diameter in nm, Zeta: Zeta potential in mV, Encap Eff.: Encapsulation efficiency in %.

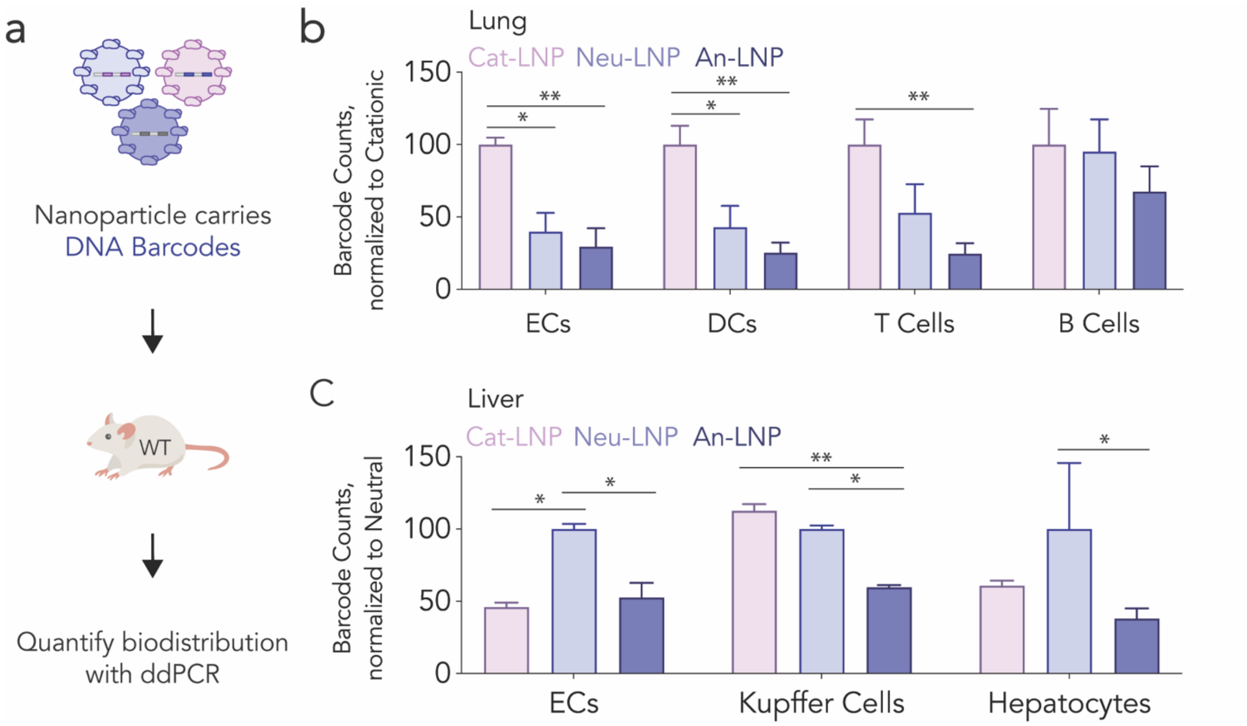


**Supplementary Figure 16**. (a) Biodistribution profile of Cat-LNP, Neu-LNP, and An-LNP. The Cat-, Neu-, and An-LNPs were individually formulated with DNA barcodes. Twenty-four hours later, distribution of each LNP was determined with ddPCR. (b, c) Biodistribution of the Cat-, Neu-, and An-LNPs was determined in (b) lung cell types and (c) liver cell types. **$P < 0.009$, *$P < 0.05$, two-way ANOVA, average +/- SEM.

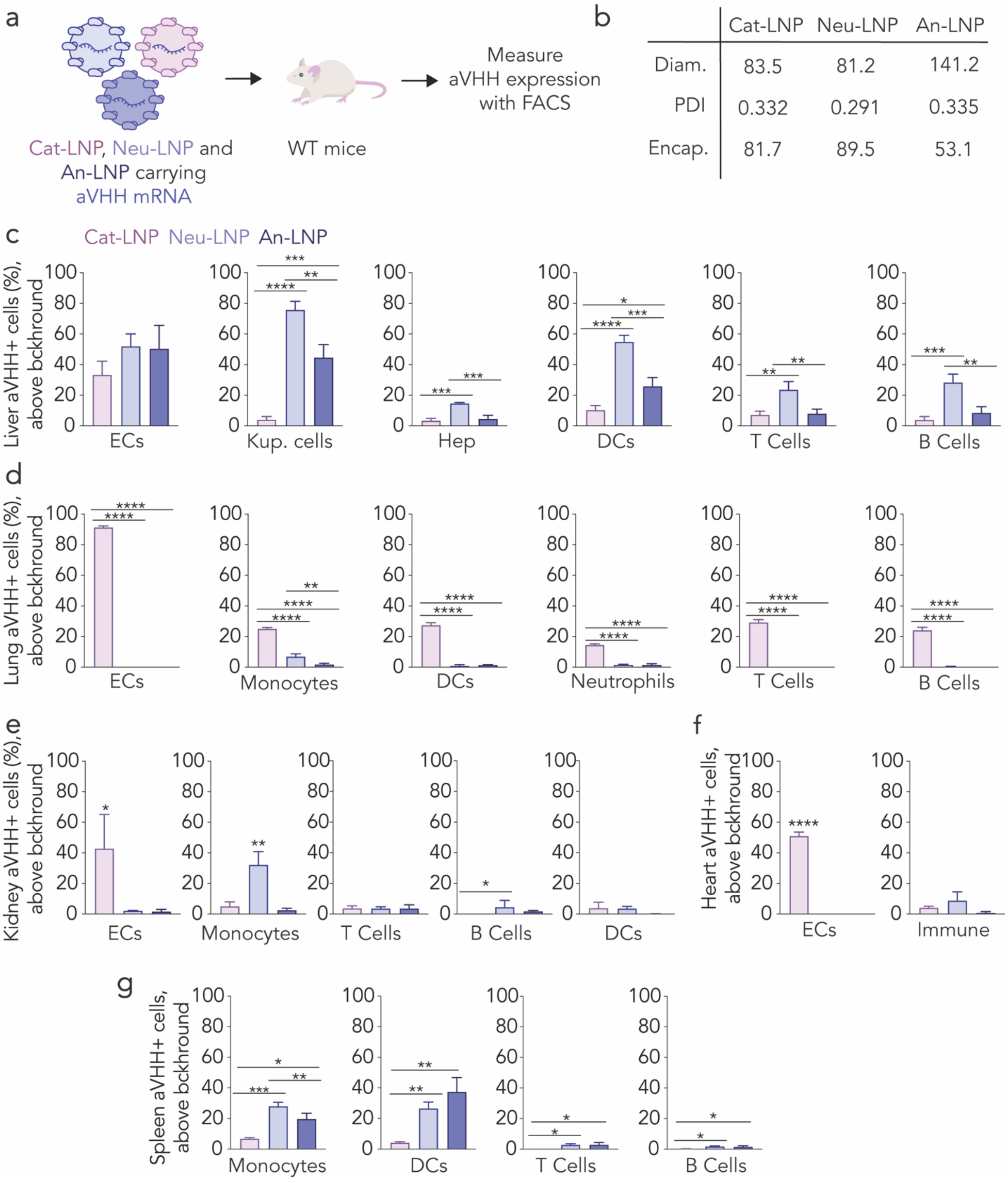
a
Cat-LNP, Neu-LNP and An-LNP carrying aVHH mRNA
WT mice
Measure aVHH expression with FACS
b
Cat-LNP Neu-LNP An-LNP
Diam. 83.5 81.2 141.2
PDI 0.332 0.291 0.335
Encap. 81.7 89.5 53.1
c
Cat-LNP Neu-LNP An-LNP
Liver aVHH+ cells (%), above bckhround
ECs
Kup. cells
Hep
DCs
T Cells
B Cells
d
Lung aVHH+ cells (%), above bckhround
ECs
Monocytes
DCs
Neutrophils
T Cells
B Cells
e
Kidney aVHH+ cells (%), above bckhround
ECs
Monocytes
T Cells
B Cells
DCs
f
Heart aVHH+ cells, above bckhround
ECs
Immune
g
Spleen aVHH+ cells, above bckhround
Monocytes
DCs
T Cells
B Cells

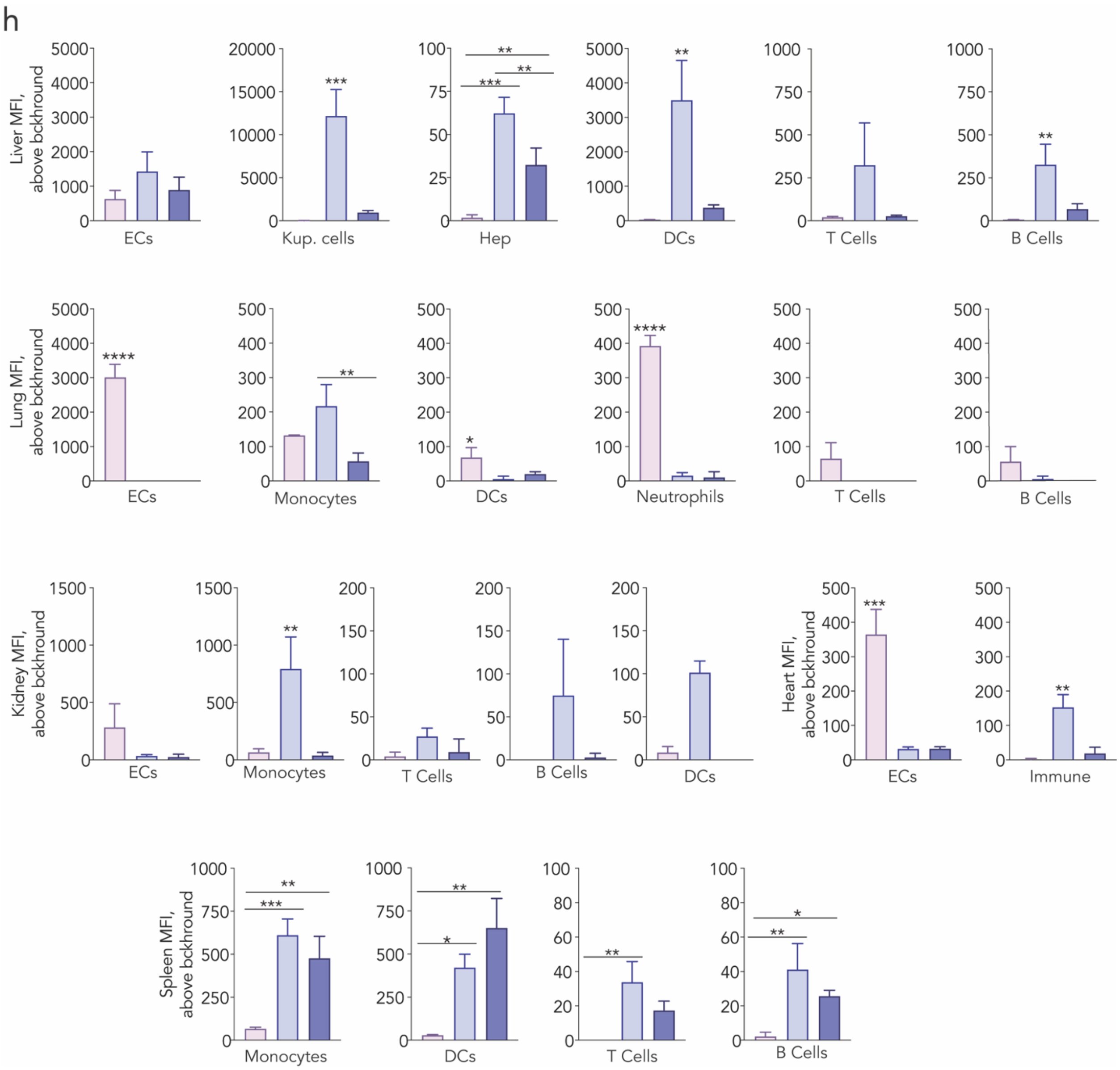


**Supplementary Figure 17.** (a) Cat-LNP, Neu-LNP and An-LNP were formulated with aVHH mRNA and administered through tail vein injection to C57BL/6 mice. Liver, lung, heart, spleen, and kidney were isolated 1 day post-injection (Diam.: Diameter (nm), PDI: polydispersity index, Encap.: Encapsulation efficiency (%)). (b) These LNPs formed small and monodisperse nanoparticles. (c) Cat-LNP de-targeted the liver, while Neu-LNP and An-LNP showed significantly higher liver tropism. (d) Cat-LNP functionally delivered aVHH mRNA to all lung cell types as compared to An-LNP and Neu-LNP. (e) Cat-LNP functionally delivered aVHH mRNA to kidney ECs. Neu-LNP showed significantly higher functional delivery to monocytes. (f) Cat-LNP significantly delivered aVHH mRNA to heart ECs. (g) Neu-LNP targeted spleen monocytes, and An-LNP targeted spleen DCs. (h) aVHH MFI measured in each cell type. ****$P < 0.0001$, ***$P <= 0.0009$, **$P <= 0.01$, *$P < 0.05$, one-way ANOVA, average +/- SD.

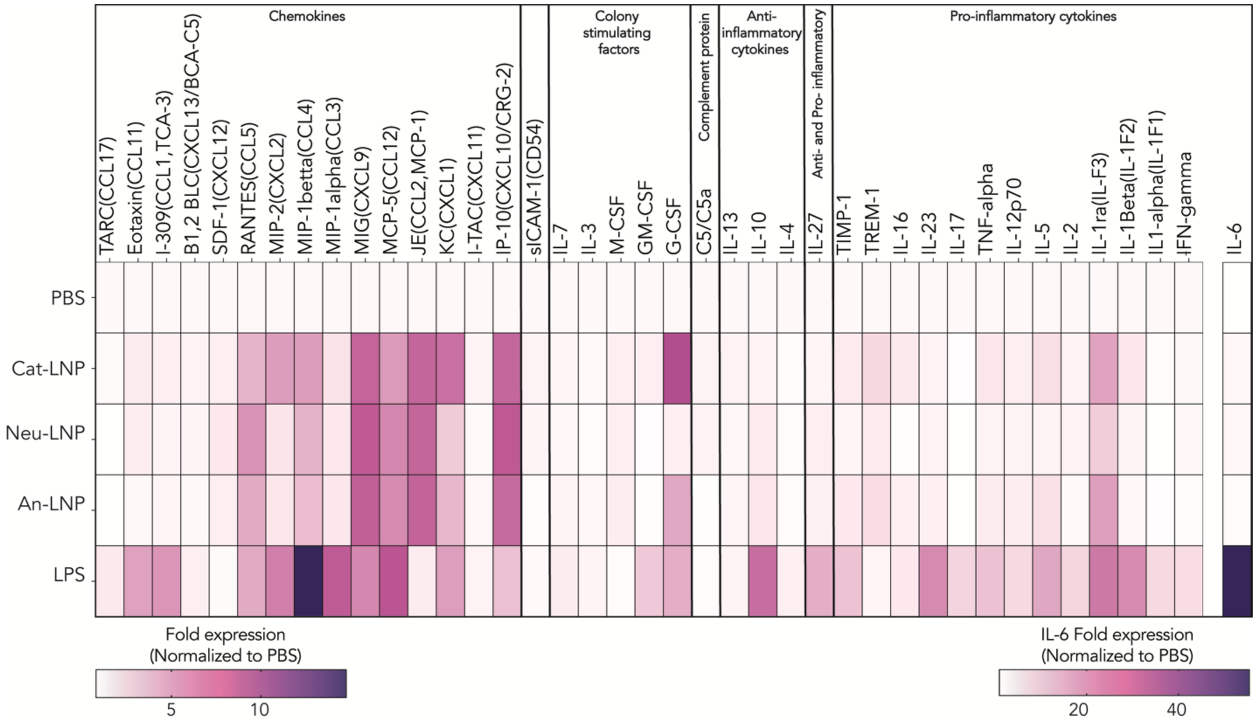


**Supplementary Figure 18**. Forty cytokines and chemokines were measured in blood serum at 6 hrs post-injection of Cat-LNP, Neu-LNP, An-LNP, PBS, and LPS at a dose of 1.3 mg/kg. Pixel intensities are normalized to PBS to report the fold expressions.

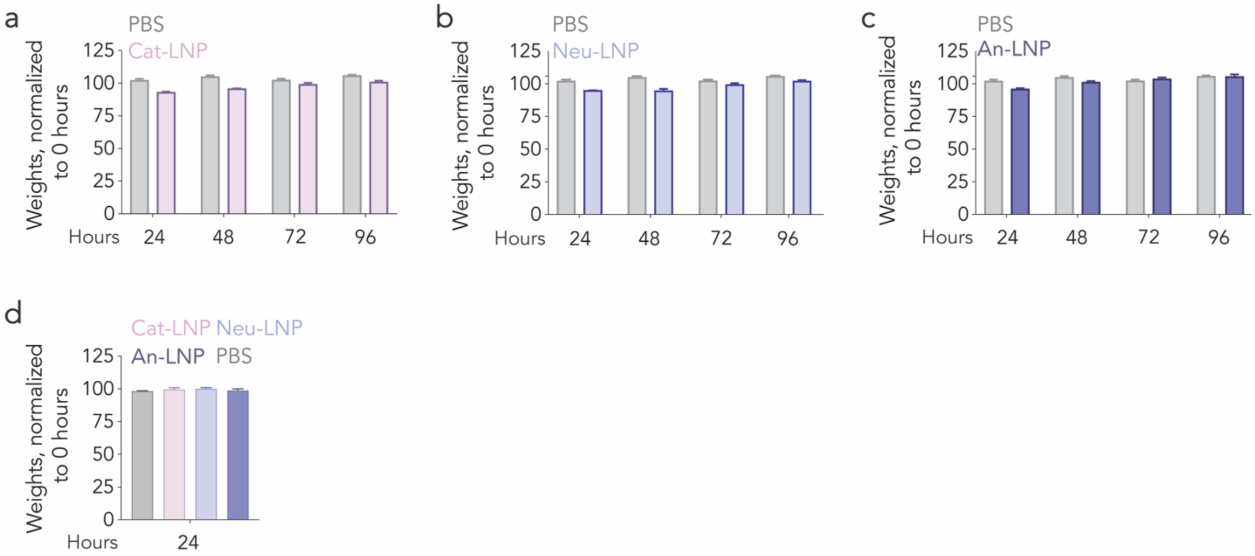


**Supplementary Figure 19.** (a–c) Mouse weights measured at 24, 48, and 96 hours after administration of PBS or Cat-, Neu-, and An-LNPs carrying Cre mRNA or (d) DNA barcodes. Average +/- SEM.

a

b

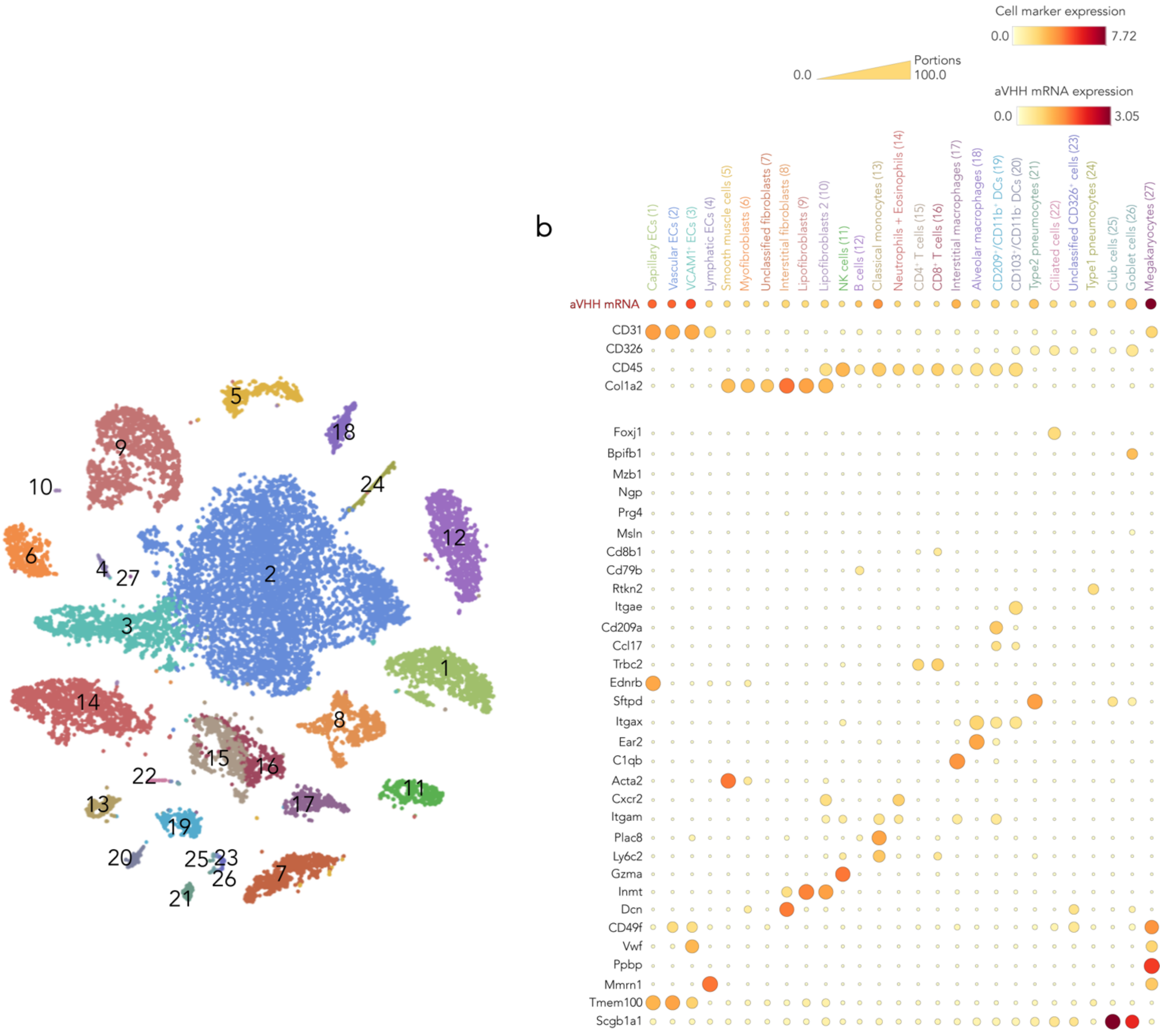

5
18
9
10
24
12
6
4
27
2
3
1
14
8
15
16
22
13
17
11
19
20
25
23
26
7
21
Cell marker expression
0.0
7.72
Portions
0.0
100.0
aVHH mRNA expression
0.0
3.05
Capillary ECs (1)
Vascular ECs (2)
VCAM1+ ECs (3)
Lymphatic ECs (4)
Smooth muscle cells (5)
Myofibroblasts (6)
Unclassified fibroblasts (7)
Interstitial fibroblasts (8)
Lipofibroblasts (9)
Lipofibroblasts 2 (10)
NK cells (11)
B cells (12)
Classical monocytes (13)
Neutrophils + Eosinophils (14)
CD4+ T cells (15)
CD8+ T cells (16)
Interstitial macrophages (17)
Alveolar macrophages (18)
CD209+/CD11b+ DCs (19)
CD103+/CD11b- DCs (20)
Type2 pneumocytes (21)
Ciliated cells (22)
Unclassified CD326+ cells (23)
Type1 pneumocytes (24)
Club cells (25)
Goblet cells (26)
Megakaryocytes (27)
aVHH mRNA
CD31
CD326
CD45
Col1a2
Foxj1
Bpifb1
Mzb1
Ngp
Prg4
Msln
Cd8b1
Cd79b
Rtkn2
Itgae
Cd209a
Ccl17
Trbc2
Ednrb
Sftpd
Itgax
Ear2
C1qb
Acta2
Cxcr2
Itgam
Plac8
Ly6c2
Gzma
Inmt
Dcn
CD49f
Vwf
Ppbp
Mmrn1
Tmem100
Scgb1a1

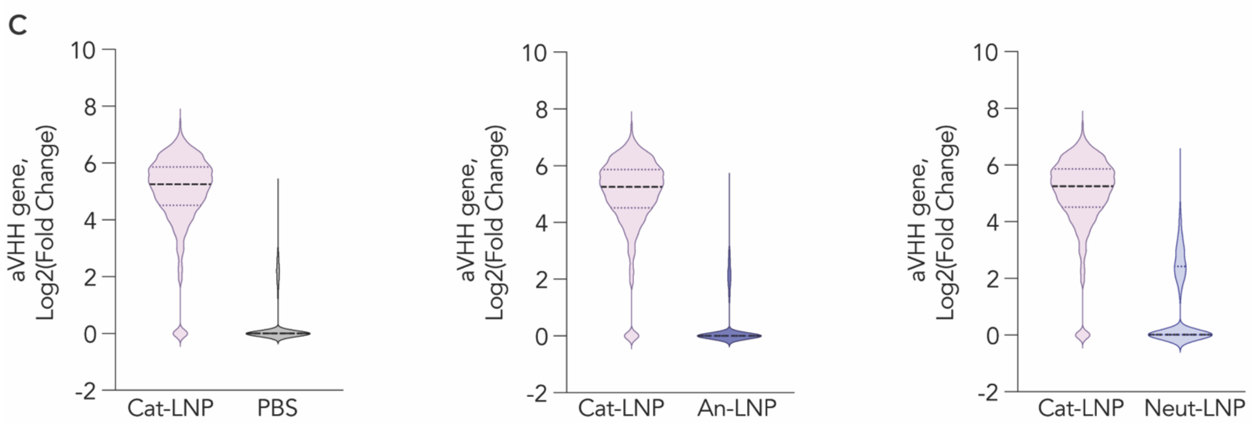


**Supplementary Figure 20.** (a) t-SNE plot showing the cells partitioned into 27 clusters. (b) Dot plot for cell marker and aVHH mRNA expressions in each cluster. (c) Cat-LNP showed about 32-fold higher aVHH mRNA expression compared to control groups (PBS, An-LNP, and Neu-LNP) in lung ECs.

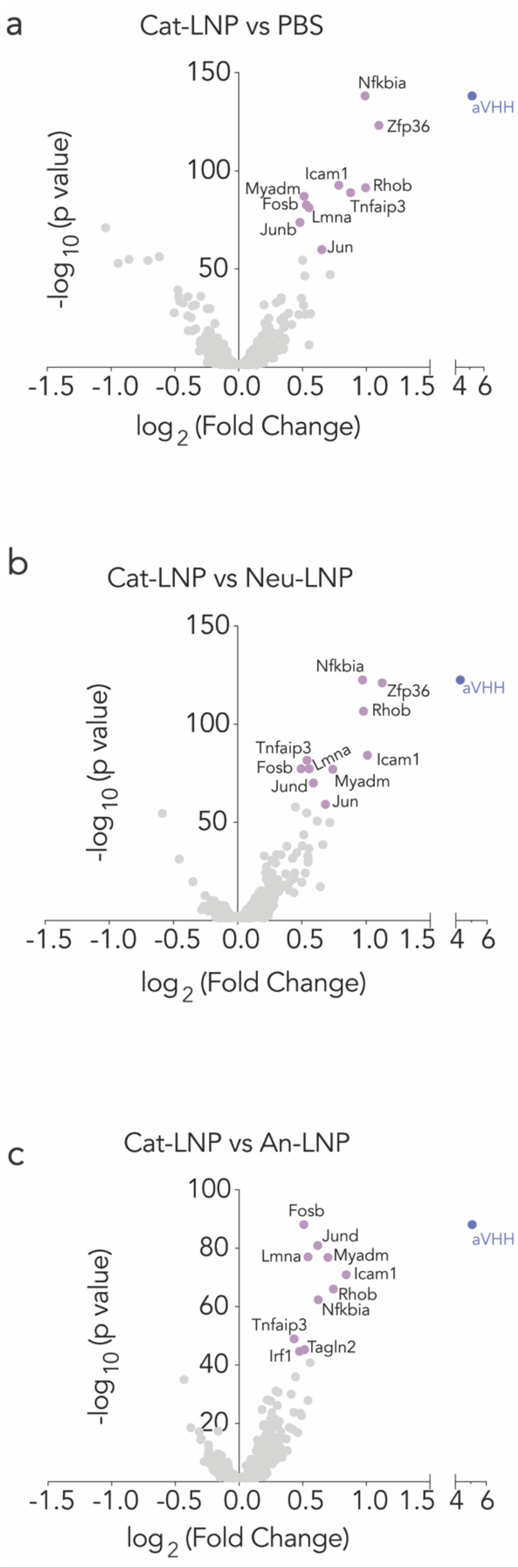
a
Cat-LNP vs PBS
150
100
50
-log 10 (p value)
Nfkbia
aVHH
Zfp36
Icam1
Myadm
Rhob
Fosb
Tnfaip3
Lmna
Junb
Jun
-1.5 -1.0 -0.5 0.0 0.5 1.0 1.5 4 6
log 2 (Fold Change)
b
Cat-LNP vs Neu-LNP
150
100
50
-log 10 (p value)
Nfkbia
Zfp36
aVHH
Rhob
Tnfaip3
Lmna
Icam1
Fosb
Jund
Myadm
Jun
-1.5 -1.0 -0.5 0.0 0.5 1.0 1.5 4 6
log 2 (Fold Change)
c
Cat-LNP vs An-LNP
100
80
60
40
20
-log 10 (p value)
Fosb
aVHH
Jund
Lmna
Myadm
Icam1
Rhob
Nfkbia
Tnfaip3
Tagln2
Irf1
-1.5 -1.0 -0.5 0.0 0.5 1.0 1.5 4 6
log 2 (Fold Change)

**Supplementary Figure 21**. Top 10 upregulated genes (purple) with Cat-LNP vs (a) PBS, (b) Neu-LNP, and (c) An-LNP P-value < 0.05.

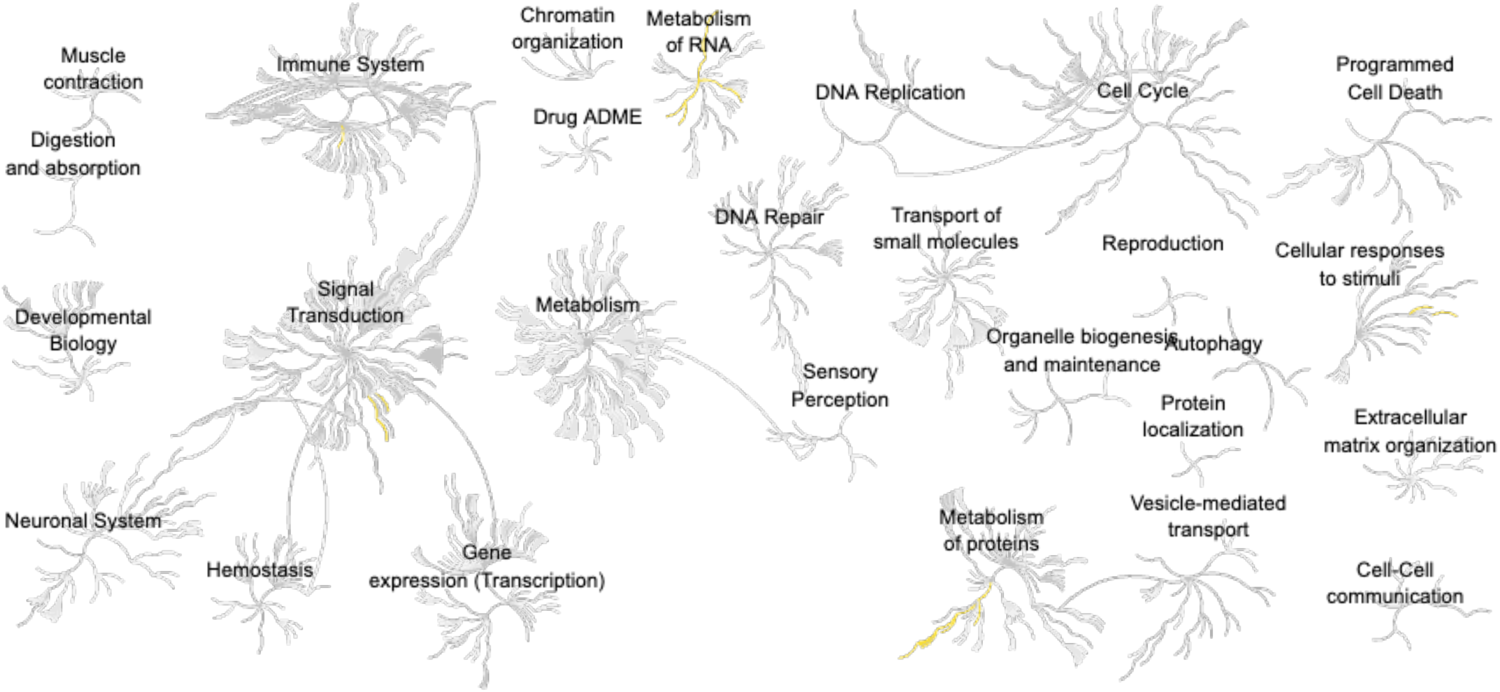


**Supplementary Figure 22.** Twenty-seven of 835 pathways were enriched in mice treated with Cat-LNP versus control groups (PBS, Neu-LNP, An-LNP), 19 of which were related to metabolism of RNA or protein, P-value < 0.001.

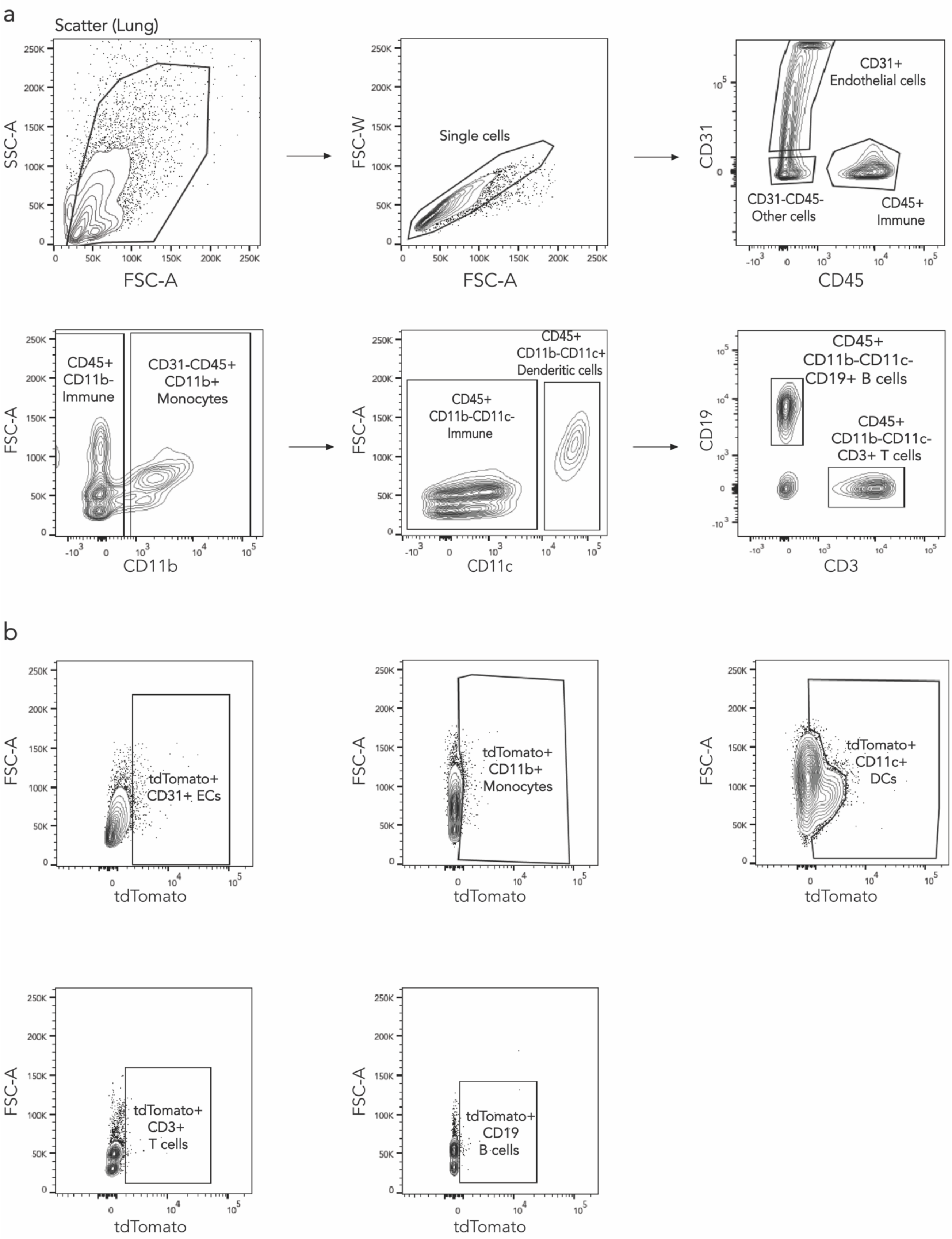
a
Scatter (Lung)
SSC-A
FSC-A
FSC-W
Single cells
CD31
CD31+
Endothelial cells
CD31-CD45-
Other cells
CD45+
Immune
CD45
FSC-A
CD45+
CD11b-
Immune
CD31-CD45+
CD11b+
Monocytes
CD11b
CD45+
CD11b-CD11c+
Denderitic cells
CD45+
CD11b-CD11c-
Immune
CD11c
CD19
CD45+
CD11b-CD11c-
CD19+ B cells
CD45+
CD11b-CD11c-
CD3+ T cells
CD3
b
tdTomato+
CD31+ ECs
tdTomato+
CD11b+
Monocytes
tdTomato+
CD11c+
DCs
tdTomato+
CD3+
T cells
tdTomato+
CD19
B cells
tdTomato

c

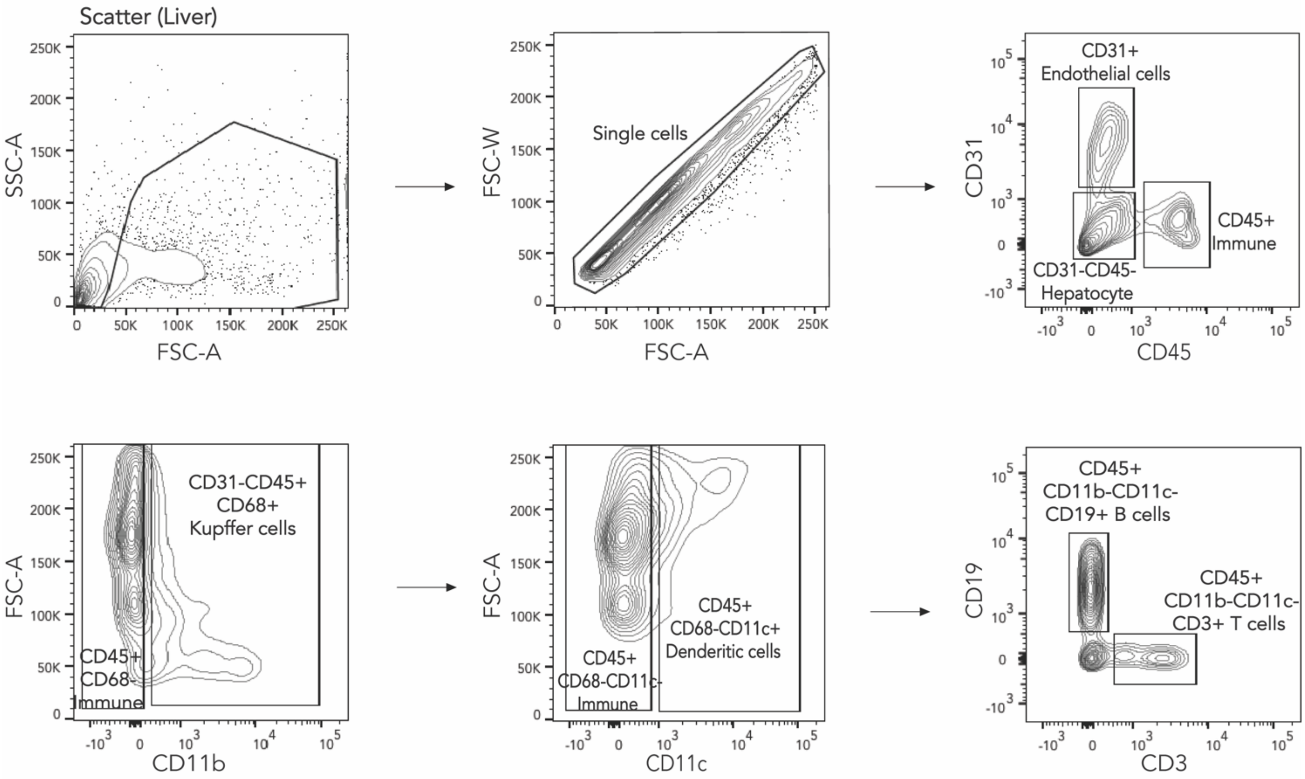
Scatter (Liver)
SSC-A
FSC-A
FSC-W
Single cells
CD31
CD31+
Endothelial cells
CD45+
Immune
CD31-CD45-
Hepatocyte
CD45
FSC-A
CD31-CD45+
CD68+
Kupffer cells
CD45+
CD68-
Immune
CD11b
CD45+
CD68-CD11c+
Denderitic cells
CD45+
CD68-CD11c-
Immune
CD11c
CD19
CD45+
CD11b-CD11c-
CD19+ B cells
CD45+
CD11b-CD11c-
CD3+ T cells
CD3

d

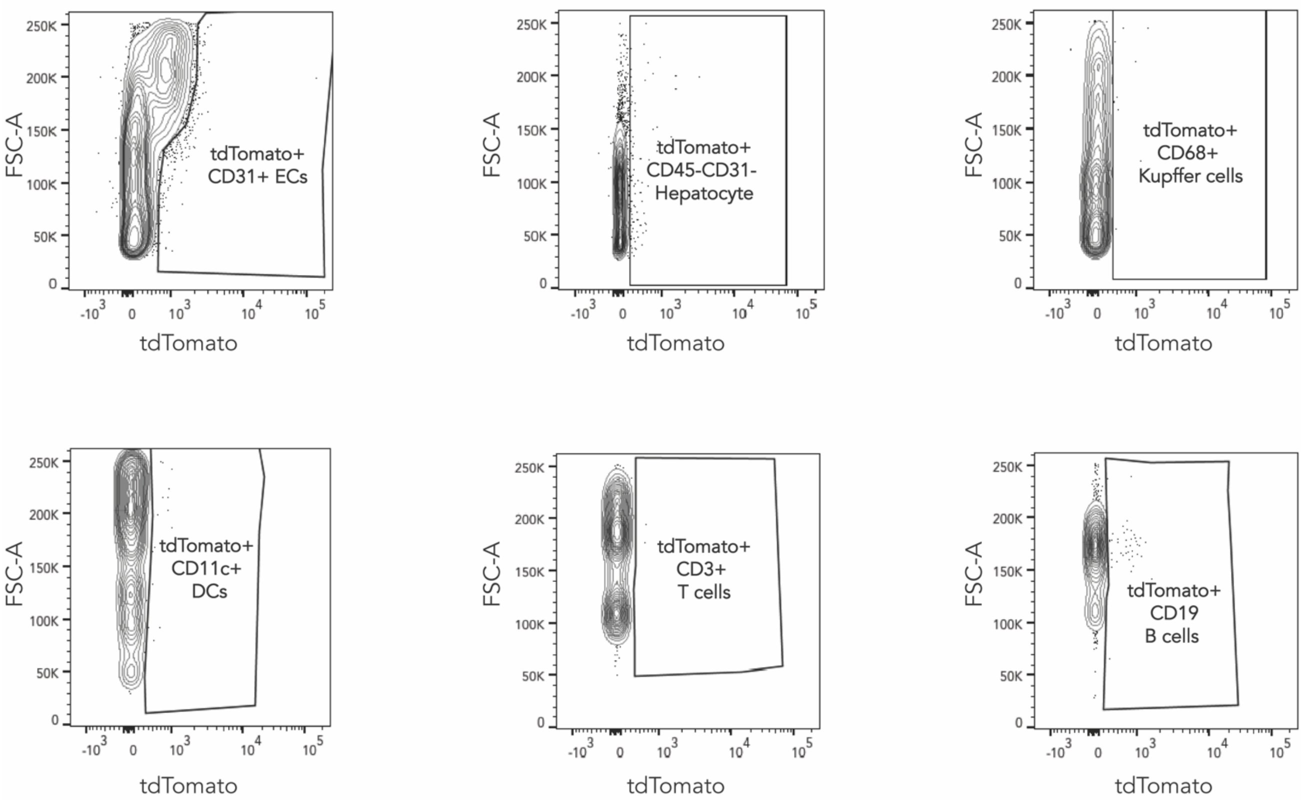
FSC-A
tdTomato+
CD31+ ECs
tdTomato+
CD45-CD31-
Hepatocyte
tdTomato+
CD68+
Kupffer cells
tdTomato+
CD11c+
DCs
tdTomato+
CD3+
T cells
tdTomato+
CD19
B cells
tdTomato

e

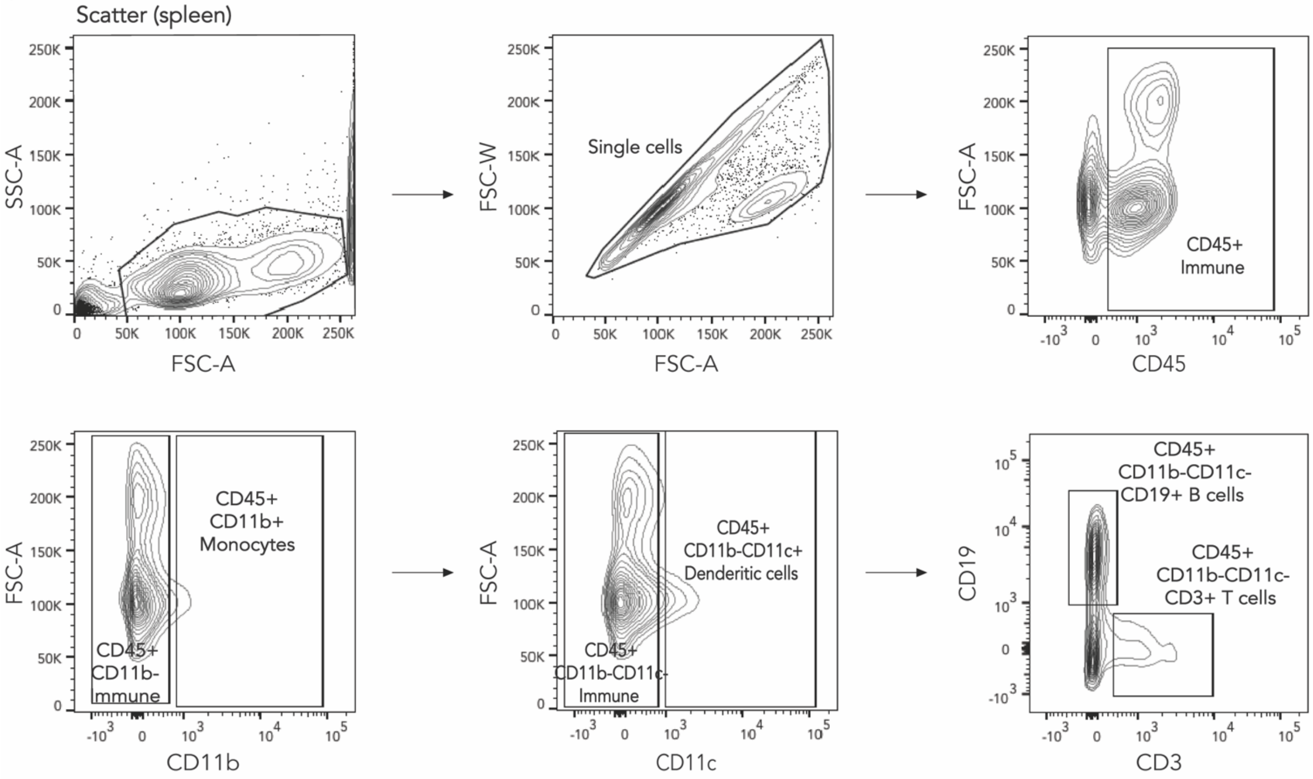
Scatter (spleen)
SSC-A
FSC-A
FSC-W
Single cells
FSC-A
CD45
CD45+
Immune
CD11b
CD45+
CD11b+
Monocytes
CD45+
CD11b-
immune
CD11c
CD45+
CD11b-CD11c+
Denderitic cells
CD45+
CD11b-CD11c-
Immune
CD19
CD3
CD45+
CD11b-CD11c-
CD19+ B cells
CD45+
CD11b-CD11c-
CD3+ T cells

f

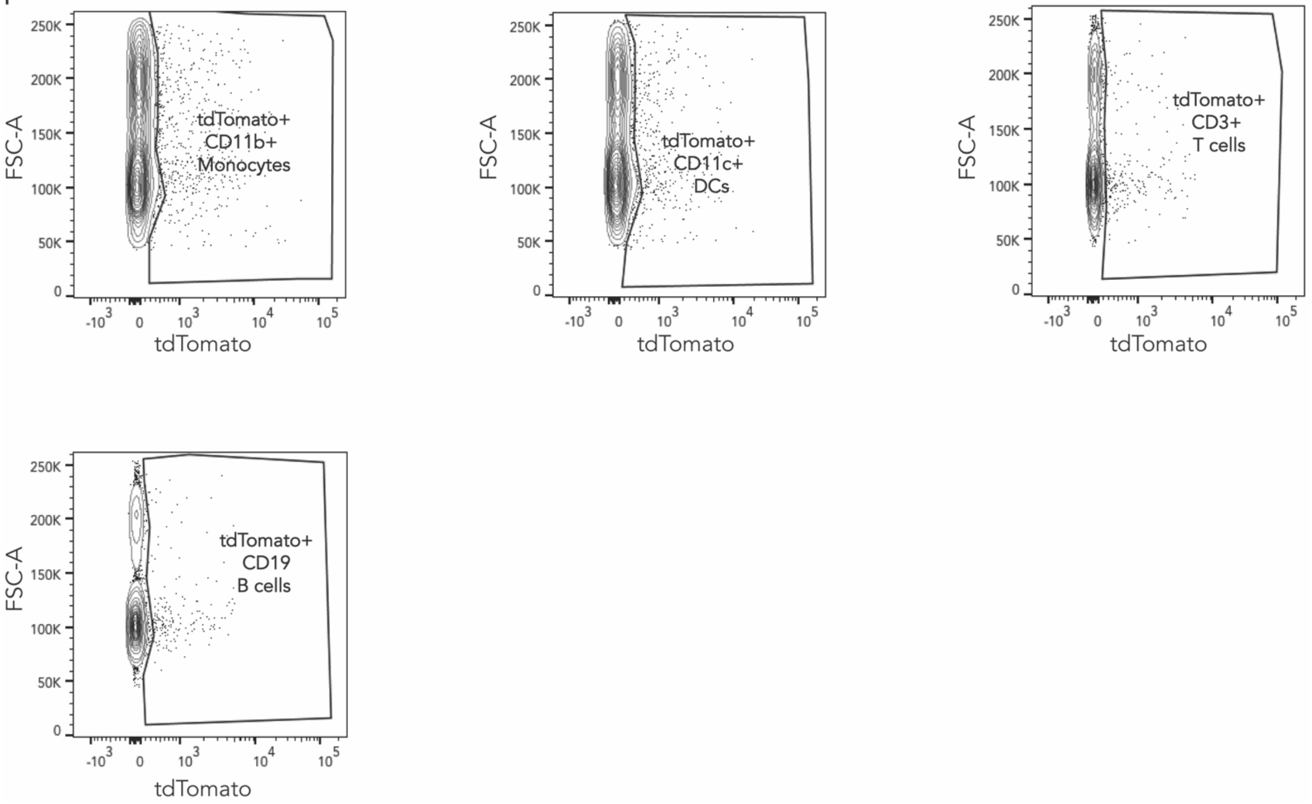
FSC-A
tdTomato
tdTomato+
CD11b+
Monocytes
tdTomato+
CD11c+
DCs
tdTomato+
CD3+
T cells
tdTomato+
CD19
B cells

g

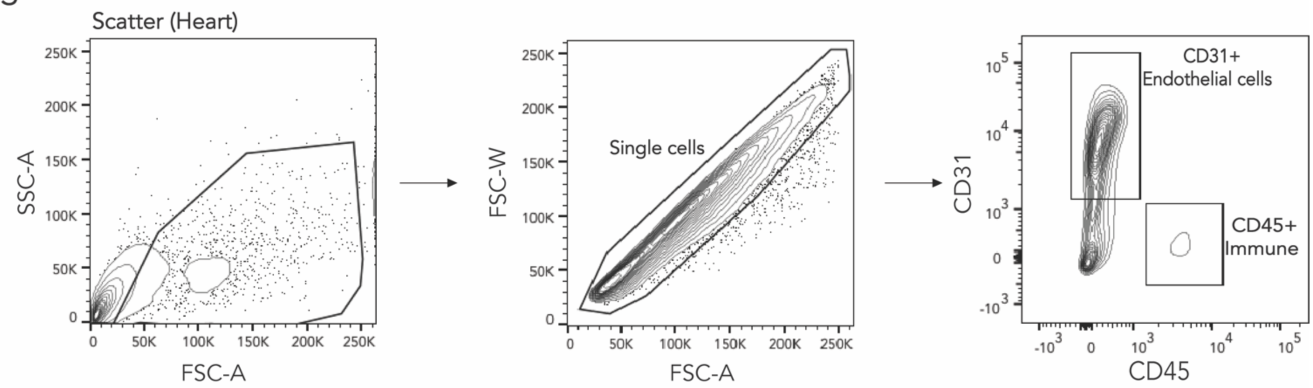

Scatter (Heart)
SSC-A
FSC-A
FSC-W
Single cells
CD31
CD45
CD31+
Endothelial cells
CD45+
Immune


h

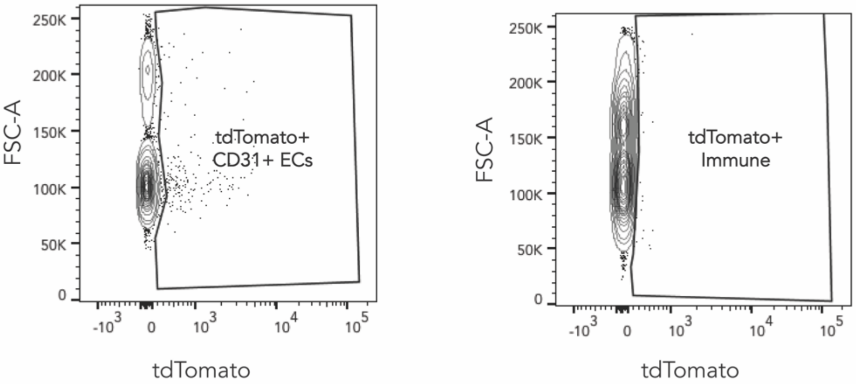

FSC-A
tdTomato
tdTomato+
CD31+ ECs
tdTomato+
Immune

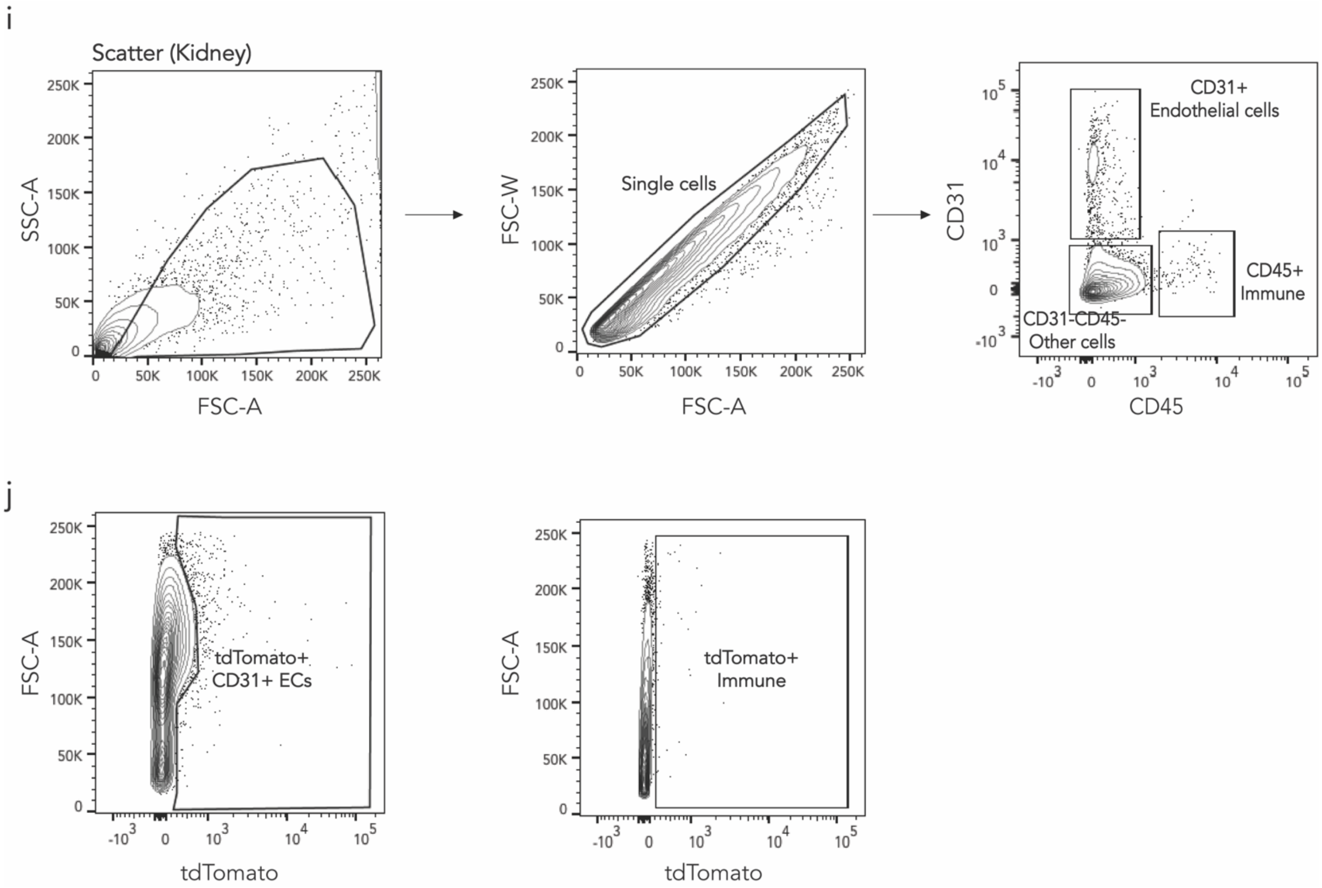


**Supplementary Figure 23**. Representative gating strategies for FACS for cell types in the (a, b) lung, (c, d) liver, (e, f) spleen, (g, h) heart, and (i, j) kidney for the screens.
